# Neuroprotective and Antioxidant Properties of Different Novel Steroid-Derived Nitrones and Oximes on Cerebral Ischemia In Vitro

**DOI:** 10.3390/ijms262311506

**Published:** 2025-11-27

**Authors:** Sara Izquierdo-Bermejo, Mourad Chioua, Dimitra Hadjipavlou-Litina, Francisco López-Muñoz, José Marco-Contelles, María Jesús Oset-Gasque

**Affiliations:** 1 Department of Biochemistry and Molecular Biology, Faculty of Pharmacy, Complutense University of Madrid, Plaza Ramón y Cajal s/n, Ciudad Universitaria, 28040 Madrid, Spain; sizqui03@ucm.es; 2 Instituto de Investigación Sanitaria del Hospital Clínico San Carlos, 28040 Madrid, Spain; 3 Faculty of Health Sciences–HM Hospitals, Camilo José Cela University, Villafranca del Castillo, 28692 Madrid, Spain; 4 Instituto Universitario de Investigación en Neuroquímica, Complutense University of Madrid, Ciudad Universitaria, 28040 Madrid, Spain; 5 Laboratory of Medicinal Chemistry, Institute of Organic Chemistry (CSIC), C/Juan de la Cierva 3, 28006 Madrid, Spain; mchioua@gmail.com; 6 Department of Pharmaceutical Chemistry, Faculty of Health Sciences, School of Pharmacy, Aristotle University of Thessaloniki, 54124 Thessaloniki, Greece; hadjipav@pharm.auth.gr; 7 HM Hospitals Health Research Institute, 28015 Madrid, Spain; 8 Neuropsychopharmacology Unit, “Hospital 12 de Octubre” Research Institute, 28041 Madrid, Spain; 9 Center for Biomedical Network Research on Rare Diseases (CIBERER), Carlos III Health Institute (ISCIII), 28029 Madrid, Spain

**Keywords:** cerebral ischemia, neuroactive steroids, neuroprotection, nitrones, oxidative stress, oximes, stroke

## Abstract

Despite the substantial global impact of ischemic stroke, current therapeutic options remain limited and only partially effective. To advance neuroprotective strategies that could improve the safety and efficacy of existing treatments while preserving brain tissue, we synthesized and evaluated seven new nitrones (**MC3**, **MC5**, **MC7**) and oximes (**MC1**, **MC2**, **MC4**, **MC6**) derived from different neuroactive steroids—ethisterone (**MC1–3**), mifepristone (**MC4–5**) and stanolone (**MC6–7**)—in an in vitro model of cerebral ischemia. Overall, these derivatives exhibited neuroprotective and antioxidant effects superior to those of the reference compounds cholesteronitrone **ChN2**, α-tert-butyl nitrone (**PBN**) and N-acetylcysteine (**NAC**). Notably, nitrones showed greater neuroprotective, anti-necrotic, and antioxidant potency than their corresponding oximes, regardless of the degree of molecular conjugation. Among them, the stanolone-derived nitrone **MC7**, which lacks conjugated double bonds, displayed the most balanced and robust profile, consistently enhancing cell viability, reducing necrotic cell death, and suppressing superoxide anion production. Consequently, **MC7** has been selected as a promising lead compound for further in vivo studies of cerebral ischemia.

## 1. Introduction

Stroke is defined as a neurological deficit attributed to acute focal damage in the central nervous system from a vascular cause [1]. It is currently the second leading cause of death and the third leading cause of disability worldwide [2]. Furthermore, its incidence continues to grow due to the rise in life expectancy in developed countries [3] and the accumulation of risk factors such as hypertension and obesity in increasingly younger populations [1,4].

Ischemic stroke is the most prevalent form, accounting for more than 85% of cases, and is characterized by a series of biochemical processes known as the ischemic cascade [5]. This type of stroke occurs when a blood vessel is occluded due to thrombus formation, leading to a reduction in blood flow to the brain [1,6]. The resulting lack of oxygen and glucose leads to energy deficits and alterations in the ionic and acid–base balance of brain cells [7,8]. These changes trigger a series of neuropathological events, such as glutamate excitotoxicity [7,8], oxidative and nitrosative stress [7,8,9], neuroinflammation [5,10] and blood–brain barrier (BBB) disruption [5,8]. All these cellular alterations promote various types of cell death, including necrosis, apoptosis, autophagy, pyroptosis, and ferroptosis [8,11].

Current therapeutic strategies for stroke treatment focus on restoring cerebral perfusion, either through pharmacological thrombolysis or mechanical thrombectomy for clot removal [1,12]. Thrombolysis involves the use of recombinant tissue plasminogen activator (rtPA) or newer derivatives, such as tenecteplase (TNK), to degrade clots by converting plasminogen into plasmin [1]. This approach has been employed since 1995 and is effective in achieving successful recanalization and reducing post-stroke sequelae in approximately 30% of patients [1]. However, rtPA and TNK have several limitations, including a narrow therapeutic window (4.5 h) [1,13], neurotoxic and pro-inflammatory side effects [1,14], and a significant risk of inducing hemorrhagic transformation [1,15]. On the other hand, thrombectomy significantly improves reperfusion rates and reduces the risk of hemorrhagic transformation, but is only effective for clots located in large vessels and requires access to specialized hospital equipment [1,10]. Moreover, the reperfusion associated with both treatments can exacerbate neuronal damage and contribute to post-stroke functional neurological deficits [16].

Given the limitations of recanalization therapies, there is significant interest in developing neuroprotective agents to prevent post-ischemic damage [17,18]. These agents aim to reduce the activation of metabolic pathways that contribute to cell death (e.g., oxidative stress, neuroinflammation) while enhancing endogenous cytoprotective responses (e.g., growth factor synthesis, stimulation of neurogenesis and angiogenesis) [8]. Combining neuroprotective strategies with current reperfusion therapies could improve the viability of brain tissue affected by ischemic injury and reduce the risk of adverse side effects [4,7]. For example, manganese porphyrins—potent catalytic antioxidants and superoxide dismutase (SOD) mimics with anti-inflammatory properties, inhibitory effects on the NF-κB pathway, and activation of *Nrf2* signaling—have shown remarkable neuroprotective potential in preclinical models of cerebral ischemia [19,20,21].

In parallel, other families of compounds have been explored for their neuroprotective efficacy, among which nitrones have attracted particular attention due to their potent free radical-scavenging properties [22]. Notably, nitrone **NXY-059**, a derivative of α-phenyl-N-tert-butylnitrone (**PBN**), reached phase III clinical trials but was ultimately discontinued due to a lack of significant efficacy in patients [1,4]. The translation of the neuroprotective potential of nitrones from laboratory studies to clinical settings has faced several challenges, including limitations of preclinical experimental models [4,23] and the inherent complexities of designing clinical trials for stroke treatment [18,23].

Although less studied, oximes also show significant neuroprotective potential, primarily through their inhibition of the MAPK c-Jun N-terminal kinase (JNK) pathway [24,25,26]. JNK plays a key role in inducing apoptosis associated with cerebral ischemia, driving caspase-3 activation and cytochrome c release from mitochondria [27,28,29], as well as promoting neuroinflammation by inducing microglial activation and increased expression of pro-inflammatory cytokines such as IL-1β, IL-6, and TNF-α [30,31]. Additionally, oximes possess antioxidant properties, scavenging free radicals and reducing lipid peroxidation, which further supports their neuroprotective efficacy [24,25]. In fact, studies have shown that oxime treatment reduces oxidative stress and neuroinflammation, enhances cerebral microcirculation, and leads to smaller infarct sizes with improved neurological outcomes in in vivo stroke models [24,25,26].

Beyond these nitrogen-based compounds, another important family of molecules with promising neuroprotective effects is that of neuroactive steroids (NS). Steroids are biologically active polycyclic molecules with distinctive physicochemical properties and diverse medical applications [32], including the treatment of cardiovascular [32,33] and neurological disorders [32,34]. Within this broad family, neuroactive steroids represent a subgroup that includes peripheral gland-derived hormones, neurosteroids synthesized by neurons and glial cells, and synthetic steroid derivatives [35,36]. Classical neurosteroids such as estrogen, progesterone, and vitamin D have been shown to protect against neuroinflammation and excitotoxicity, exert anti-apoptotic and antioxidant effects, and promote angiogenesis and neurogenesis [32,35,36,37,38,39]. As a result, they hold substantial therapeutic promise for reducing brain damage associated with ischemic stroke, a notion supported by in vitro studies showing that these compounds promote autophagy and inhibit apoptosis in neuronal cell cultures [40,41] as well as by in vivo studies demonstrating that their administration consistently decreases infarct volume and preserves motor function [35,36].

Therefore, combining the neuroprotective properties of nitrones and oximes with the well-documented benefits of neuroactive steroids represents a promising strategy for developing new therapeutic candidates for ischemic stroke. Our research group has long focused on the design, synthesis, and characterization of novel steroid-based compounds with neuroprotective potential [42,43,44]. In previous work, we synthesized cholesteronitrone **ChN2** [42,43], derived from the structure of **olesoxime** (cholest-4-en-3-one), a cholesterol-based molecule initially developed in 2007 as a therapeutic candidate for amyotrophic lateral sclerosis (ALS) and shown to possess neuroprotective properties in preclinical models of various neurodegenerative diseases [45,46]. **ChN2** demonstrated strong neuroprotective and antioxidant effects in both in vitro and in vivo models of cerebral ischemia [42,43,44].

**Figure 1:**
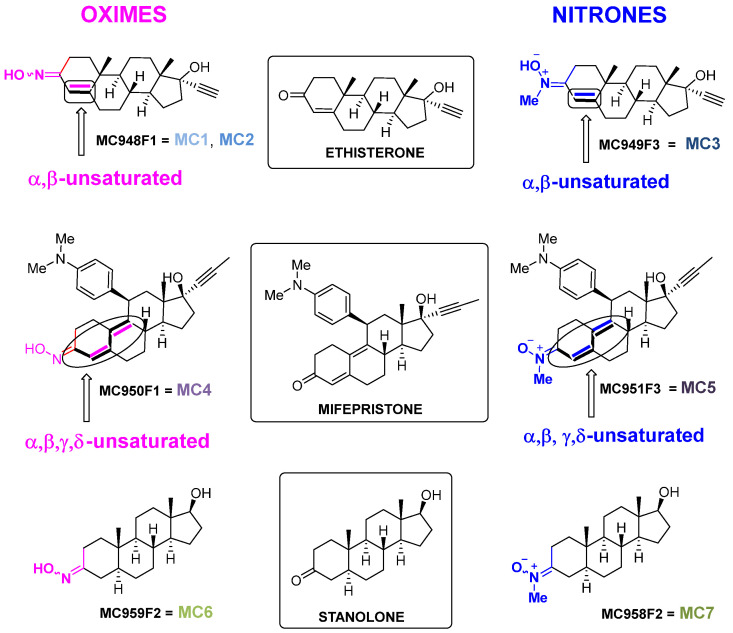
Structure of ethisterone, mifepristone, stanolone, and their corresponding oxime and nitrone derivatives.

On this basis, the present study explores the synthesis and biological evaluation of seven new steroid-derived molecules: three nitrones and four oximes (Figure 1), focusing on their antioxidant potential and neuroprotective efficacy in mitigating necrosis and apoptosis associated with ischemic injury. The steroidal scaffolds used as precursors for the synthesis of these compounds include ethisterone, mifepristone, and stanolone (Figure 1).

(1) **Ethisterone** (Figure 1), also known as ethinyltestosterone, pregneninolone, and anhydrohydroxyprogesterone, is a synthetic progestogen (i.e., an agonist of the progesterone receptor) historically used as a progestin medication to treat gynecological disorders [47,48,49,50]. Although no longer in clinical use, ethisterone’s steroidal framework provided a valuable starting point for chemical modifications. From ethisterone, two oxime derivatives (MC948F1 = **MC1**, **MC2**) and one nitrone derivative (MC949F3 = **MC3**) were synthesized (**group 1**). All three are known compounds that have been prepared as previously described [51,52], showing analytical and spectroscopic data consistent with published values (see Supporting Information) [49,51,52]. Notably, the oxime derivatives exhibited distinct E/Z isomer ratios, with **MC1** showing a 2:1 ratio and **MC2** a 4:1 ratio.
(2) **Mifepristone** (Figure 1), also known as RU-486, is an antiprogestogen (i.e., it blocks the effects of progesterone) widely recognized for its medical use in pregnancy termination and miscarriage management [53]. From mifepristone, one oxime derivative (MC950F1 = **MC4**) and one nitrone derivative (MC951F3 = **MC5**) were synthesized (**group 2**). While **MC4** had been previously described [50], **MC5** was synthesized here for the first time, with analytical and spectroscopic data confirming its structure (see Supporting Information).
(3) **Stanolone** (Figure 1), also known as dihydrotestosterone (DHT), is an androgen and anabolic steroid used clinically to treat testosterone deficiency and other related conditions [54]. From stanolone, one oxime derivative (MC959F2 = **MC6**) [48,55] and one nitrone derivative (MC958F2 = **MC7**) were synthesized (**group 3**) [49]. Both compounds are known and were prepared as described in the literature, with analytical and spectroscopic data consistent with previously reported values (see Supporting Information) [48,49,55].

As shown in Figure 1, the oxime and nitrone derivatives of ethisterone, mifepristone, and stanolone exhibit varying degrees of conjugation between unsaturated double bonds and their oxime or N-methyl nitrone moieties. In the case of ethisterone oxime and nitrone derivatives (**MC1**, **MC2** and **MC3**), a single conjugated double bond is present in ring A, specifically between positions C4 and C5. For mifepristone oxime and nitrone derivatives (**MC4** and **MC5**), two conjugated double bonds are observed: one in ring A (C4–C5) and an additional conjugated double bond in ring B (C9–C10). By contrast, stanolone oxime and nitrone derivatives (**MC6** and **MC7**) lack any conjugated double bonds in their structures.

This design and selection of the precursors aims to investigate the differential impact on the neuroprotective and antioxidant effects of these compounds by evaluating:

(a) A single conjugated double bond associated with the oxime (or nitrone) moiety, as observed in the ethisterone derivatives (**MC1**, **MC2**, and **MC3**).
(b) Two non-linear conjugated double bonds associated with the oxime (or nitrone) moiety, as observed in the mifepristone derivatives (**MC4** and **MC5**).
(c) The absence of conjugation in the oxime (or nitrone) moiety, as observed in the stanolone derivatives (**MC6** and **MC7**).

In addition, comparing nitrones ([R_1_R_2_C=N^+^(Me)O^−^]) and oximes (R_1_R_2_C=NOH) within the same steroid-based frameworks—neuroactive steroidal nitrones (NSNs) and oximes (NSOs)—allow for the evaluation of their respective efficiencies in trapping reactive oxygen species (ROS) and protecting from cell death. This comparison is particularly relevant given that all compounds exhibit good brain permeability, ensuring their potential suitability for mitigating oxidative stress in the context of the ischemic cascade.

The neuroprotective and antioxidant capacity of the seven compounds (**MC1–7**) was compared to that of **ChN2** and two standards with proven efficacy, namely, **PBN** and N-acetylcysteine (**NAC**). **PBN** was selected due to its extensive characterization in preclinical models and its role as the precursor of the first nitrone to reach phase III clinical trials [1,4], whereas **NAC** was included for its well-established antioxidant, anti-inflammatory, and mitochondrial-protective properties, as well as its documented neuroprotective effects in both experimental and clinical studies of cerebral ischemia [56,57,58,59,60,61,62,63].

## 2. Results

### 2.1. Basal Neurotoxicity of NSNs, NSOs, ChN2, PBN and NAC

**Figure 2:**
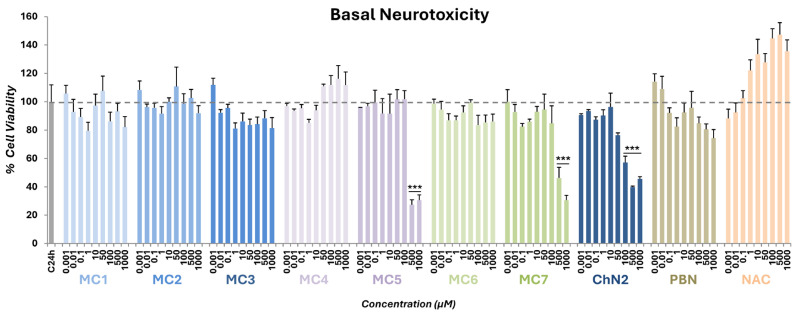
Effect of **MC1–7**, **ChN2**, **PBN**, and **NAC** on SH-SY5Y human neuroblastoma cell viability under basal conditions. The bars represent the % of cell viability at the indicated concentrations of the compounds. The viability of untreated cells (C24h) was set at 100% (100 ± 11.9%; mean ± SEM) (dotted line). Data are shown as means ± SEM of three independent experiments performed in triplicate. The statistical analysis (one-way ANOVA) reveals significant differences in viability compared to C24h (*** *p* < 0.001). Statistical analysis of results exceeding 100% is not displayed.

As a preliminary step in evaluating the neuroprotective effects of the test compounds in the experimental model of ischemia, we first assessed their basal neurotoxicity in the absence of any toxic insult using the XTT assay. As shown in Figure 2, nitrones **MC5** and **MC7** exhibited significant neurotoxicity at the highest concentrations, with a 50–70% loss of cell viability observed at 500 and 1000 μM. **ChN2**, in line with previously published data [44], showed a significant decrease in cell death starting at 100 μM. Therefore, the inherent neurotoxicity of these three compounds was taken into account when evaluating the results of the neuroprotection assays. In contrast, no substantial neurotoxic effects were observed for the remaining test compounds, as well as for **PBN** and **NAC**, at any of the concentrations tested.

### 2.2. Neuroprotective Profiles of NSNs, NSOs, ChN2, PBN and NAC in a Cellular Model of Cerebral Ischemia

#### 2.2.1. Effect on Cell Viability

After analyzing the basal neurotoxicity of the compounds, we proceeded to investigate their neuroprotective capacity under the conditions of the in vitro ischemia–reperfusion (I/R) model, which simulates the recanalization process. This is particularly relevant as it represents the optimal timeframe for the intervention of neuroprotective agents in the treatment of ischemic stroke [17,18]. The XTT assay was used to evaluate cellular metabolic capacity after exposure to the injury model and subsequent treatment with the study compounds.

**Figure 3:**
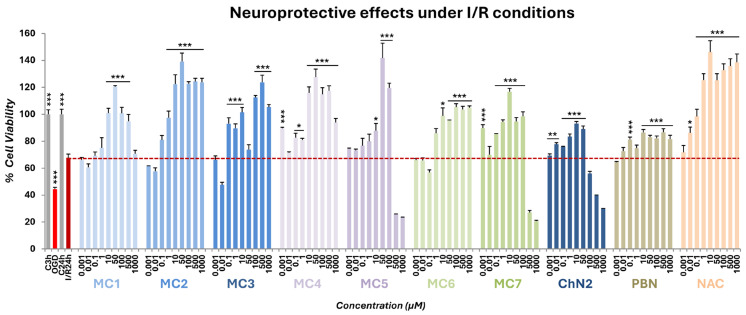
Neuroprotective effects of **MC1–7**, **ChN2**, **PBN**, and **NAC** against the reduction in metabolic capacity induced by oxygen–glucose deprivation (OGD) (3 h) followed by 24 h of oxygen and glucose resupply (I/R, dotted line) in SH-SY5Y human neuroblastoma cells. C denotes control cells maintained under standard conditions of oxygen and glucose. C3h corresponds to the control condition for OGD, and C24h corresponds to the control condition for I/R. The bars represent the % of cell viability at the indicated concentrations of the compounds. Data are shown as means ± SEM of three independent experiments performed in triplicate. The statistical analysis (one-way ANOVA followed by Holm–Sidak post hoc test) reveals significant differences in viability compared to the I/R condition (* *p* < 0.05; ** *p* < 0.01; *** *p* < 0.001).

OGD exposure resulted in a significant reduction in cell viability (44.45 ± 1.30%, mean ± SEM; n = 6; *p* < 0.001 vs. C3h, one-way ANOVA test), which showed partial recovery after 24 h of reperfusion (67.75 ± 2.67%, mean ± SEM; n = 6; *p* < 0.001 vs. C24h, one-way ANOVA test) (Figure 3).

The seven test compounds displayed neuroprotective effects by mitigating the I/R-induced decline in metabolic activity, with efficacy varying across different concentration ranges (Figure 3). Overall, the neuroprotective capacity of **MC1–7** was superior to that of **ChN2** and **PBN** and, in some cases, comparable to that of **NAC** (Figure 3).

**Figure 4:**
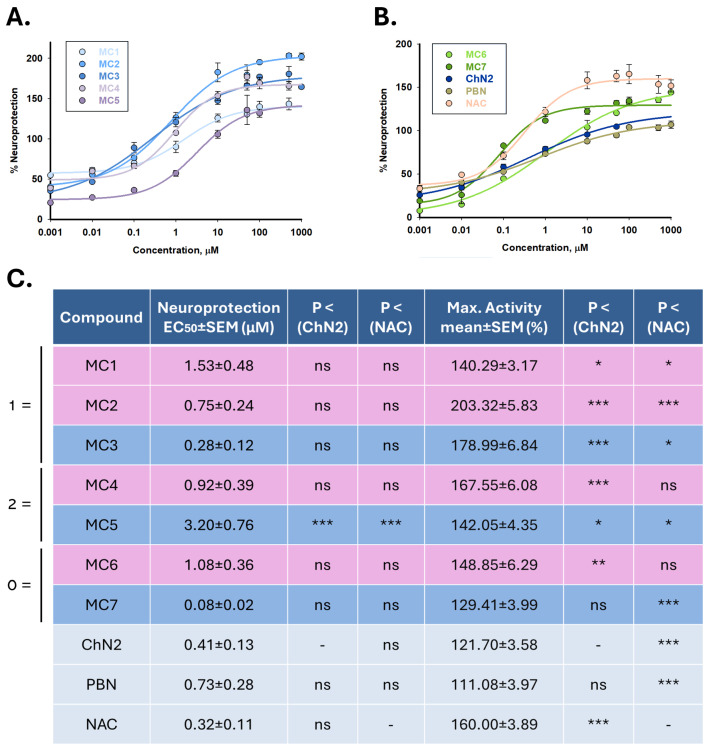
Neuroprotective effects of **MC1–7**, **ChN2**, **PBN**, and **NAC** in SH-SY5Y cells following I/R exposure. (**A**,**B**) Concentration–response curves showing the percentage of neuroprotection of each compound at the indicated concentrations. The curves were fitted using non-linear weighted regression analysis to estimate the effective dose 50 (EC_50_) and maximal neuroprotective activity (MNA) values. Data represent the means ± SEM of three independent experiments, each performed in triplicate. (**C**) EC_50_ and MNA values of the tested compounds. Rows highlighted in pink correspond to oximes, those in dark blue to nitrones, and the remaining to the three reference compounds. The structural labels 0 =, 1 =, and 2 = indicate the number of conjugated double bonds in the molecules: 0 = no double bonds, 1 = one double bond, 2 = two double bonds. Columns “*p* < (ChN2)” and “*p* < (NAC)” indicate the significance of the difference relative to **ChN2** and **NAC**, respectively. * *p* < 0.05, ** *p* < 0.01, and *** *p* < 0.001; ns: not statistically significant (one-way ANOVA with Holm–Sidak post hoc test).

To further investigate the pharmacodynamic properties of the tested compounds, we determined their EC_50_ values and maximal neuroprotective activity (MNA) as indicators of potency and efficacy, respectively (Figure 4). Under I/R conditions, the EC_50_ values of the compounds, from lowest to highest, were as follows: **MC7** ≤ **MC3** ≤ **NAC** ≤ **ChN2** ≤ **PBN** ≤ **MC2** ≤ **MC4** ≤ **MC6** ≤ **MC1** ≤ **MC5** (Figure 4C). These results indicate that the new nitrones and oximes exhibit similar potency to that of the reference compounds, with the exception of nitrone **MC5**, which displayed a significantly higher EC_50_, indicating lower potency. In contrast, the MNA values followed this descending order: **MC2** > **MC3** ≥ **MC4** ≥ **NAC** ≥ **MC6** ≥ **MC5** ≥ **MC1** ≥ **MC7** ≥ **ChN2** ≥ **PBN** (Figure 4C). Accordingly, oxime **MC2** exhibited significantly higher maximal neuroprotective activity compared to all other compounds, among which no statistically significant differences were found. These findings reveal divergent trends in the pharmacodynamic properties of the seven new compounds: while nitrones tend to show greater potency (i.e., lower EC_50_ values) than their corresponding oximes—with the exception of **MC5**—, they generally achieve lower maximal neuroprotective activity. Moreover, a higher degree of conjugation within the molecular structure did not correlate with increased potency; in fact, the opposite trend was observed. Group 3 nitrones (lacking double bonds) were the most potent, followed by group 1 nitrones (bearing a single conjugated double bond), whereas group 2 nitrones (with two conjugated double bonds) were the least potent. The number of double bonds also influenced efficacy: group 1 compounds exhibited the highest MNA values, followed by group 2 compounds, whereas non-conjugated compounds showed the lowest efficacy.

Taken together, these results demonstrate that nitrones **MC3** and **MC7** markedly enhance the neuroprotective properties of the reference nitrone **ChN2**, exhibiting lower EC_50_ and higher MNA values. In contrast, the oximes display higher EC_50_ values than both **ChN2** and **NAC**, although they show slightly greater efficacy. Among oximes, **MC2** emerged as the most promising candidate, while among the nitrones, **MC3** exhibited a favorable balance between potency and efficacy (Figure S1A,A’, Supplementary Material). Nevertheless, **MC7** remained the most potent nitrone overall, despite showing some cytotoxicity at higher concentrations.

#### 2.2.2. Effects on Necrotic Cell Death

Building upon the previous findings, we next assessed the test compounds’ anti-necrotic effects. Necrosis is a form of cell death characterized by cell swelling, disruption of intracellular organelles, and ultimately, breakdown of the plasma membrane [64]. Lactate dehydrogenase (LDH), a soluble cytosolic enzyme, readily diffuses through compromised membranes. Therefore, comparing LDH activity in the culture medium to its intracellular activity provides an estimate of the extent of necrotic cell death following I/R exposure [64].

**Figure 5:**
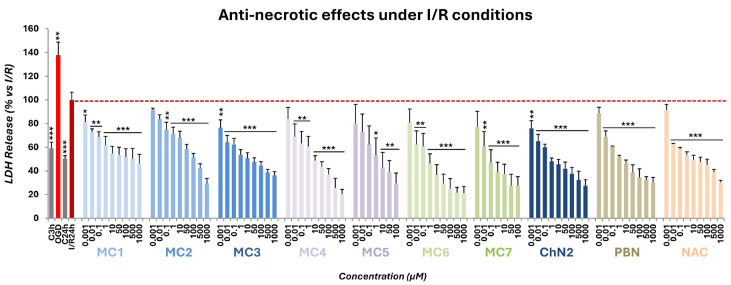
Anti-necrotic effects of **MC1–7**, **ChN2**, **PBN**, and **NAC** in SH-SY5Y human neuroblastoma cells following OGD (3 h) and subsequent I/R (24 h) (dotted line). C denotes control cells maintained under standard conditions of oxygen and glucose. C3h corresponds to the control condition for OGD, and C24h corresponds to the control condition for I/R. The bars represent the % of lactate dehydrogenase (LDH) released under the indicated experimental conditions. Data are shown as means ± SEM of three independent experiments, each performed in triplicate. The statistical analysis (one-way ANOVA followed by Holm–Sidak post hoc test) reveals significant differences in LDH release compared to the I/R condition (* *p* < 0.05; ** *p* < 0.01; *** *p* < 0.001).

As shown in Figure 5, the percentage of LDH release following I/R exposure was set as 100%, and the values obtained from OGD (137.90 ± 10.86%) and their corresponding controls at 3 h and 24 h were normalized accordingly (59.09 ± 5.27% for C3h and 50.50 ± 2.42% for C24h; mean ± SEM; n = 6). Based on these data, the net necrotic effects of OGD and I/R relative to their respective controls were 78.81 ± 8.06% and 49.50 ± 4.47%, respectively, indicating that I/R significantly reduced the necrotic effect induced by OGD by 29.31 ± 6.27% (*p* < 0.01, one-way ANOVA; Figure 5).

All seven test compounds significantly reduced LDH release in a concentration-dependent manner, with the exception of the lowest concentrations of **MC4**, **MC6**, and **MC7** (0.001 μM), as well as **MC2** (0.001–0.01 μM) and **MC5** (0.001–0.1 μM) (Figure 5). Overall, the anti-necrotic effects of **MC1–7** were comparable to those observed for the three reference compounds (Figure 5).

**Figure 6:**
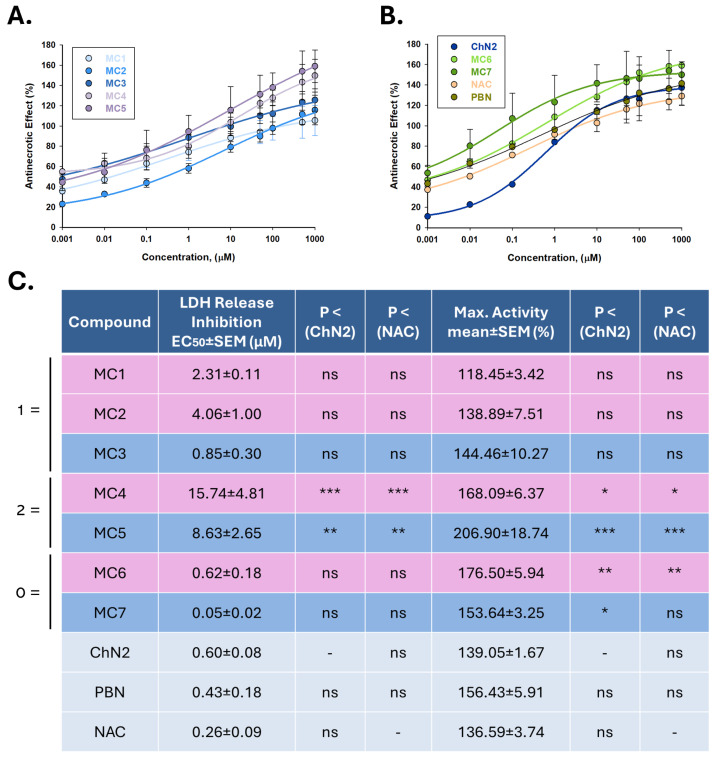
Anti-necrotic effects of **MC1–7**, **ChN2**, **PBN**, and **NAC** in SH-SY5Y cells following I/R exposure. (**A**,**B**) Concentration–response curves showing the percentage of anti-necrotic activity of each compound at the indicated concentrations. The curves were fitted using non-linear weighted regression analysis to estimate the effective dose 50 (EC_50_) and maximal anti-necrotic activity (MANA) values. Data represent the means ± SEM of three independent experiments, each performed in triplicate. (**C**) EC_50_ and MANA values of the tested compounds. Rows highlighted in pink correspond to oximes, those in dark blue to nitrones, and the remaining to the three reference compounds. The structural labels 0 =, 1 =, and 2 = indicate the number of conjugated double bonds in the molecules: 0 = no double bonds, 1 = one double bond, 2 = two double bonds. Columns “*p* < (ChN2)” and “*p* < (NAC)” indicate the significance of the difference relative to **ChN2** and **NAC**, respectively. * *p* < 0.05, ** *p* < 0.01, and *** *p* < 0.001; ns: not statistically significant (one-way ANOVA with Holm–Sidak post hoc test).

Concentration–response curves ranging from 0.001 to 1000 μM were fitted using non-linear regression analyses to calculate EC_50_ and maximal anti-necrotic activity (MANA) values (Figure 6). The EC_50_ values of the compounds, from lowest to highest, were as follows: **MC7** ≤ **NAC** ≤ **PBN** ≤ **ChN2** ≤ **MC6** ≤ **MC3** ≤ **MC1** ≤ **MC2** ≤ **MC5** ≤ **MC4** (Figure 6C). These results indicate that most of the newly synthesized nitrones and oximes display anti-necrotic potency comparable to that of the reference compounds. However, the two mifepristone-derived compounds (**MC4** and **MC5**) were the exceptions, exhibiting significantly higher EC_50_ values than the other oximes and nitrones, respectively. Conversely, the MANA values followed this descending order: **MC5** ≥ **MC6** ≥ **MC4** ≥ **PBN** ≥ **MC7** ≥ **MC3** ≥ **ChN2** ≥ **MC2** ≥ **NAC** ≥ **MC1** (Figure 6C). In this case, the mifepristone- (**MC4** and **MC5**) and stanolone-derived (**MC6** and **MC7**) compounds showed significantly greater maximal anti-necrotic activity than both **ChN2** and the ethisterone-based compounds. These findings suggest that the degree of conjugation within the molecular scaffold strongly influences the anti-necrotic effects of these new nitrones and oximes, being most favorable in **MC6** and **MC7**, which lack conjugated double bonds and demonstrated the most balanced profiles in terms of both potency and efficacy.

When comparing nitrone and oxime counterparts, nitrones from all three structural groups (**MC3**, **MC5** and **MC7**) were consistently more potent and generally showed more efficacy than their corresponding oximes, although these differences did not reach statistical significance within group 3 (**MC6** and **MC7**), which overall exhibited the lowest EC_50_ values (Figure S1B,B’, Supplementary Material). Altogether, these results indicate that nitrones possess superior anti-necrotic properties compared with oximes, with the non-conjugated nitrone **MC7** showing the greatest activity—comparable to that of **NAC** and **ChN2**—followed by nitrone **MC3**.

#### 2.2.3. Effects on Apoptotic Cell Death

To gain a more complete understanding of the neuroprotective profile of the test compounds, their anti-apoptotic effects were examined. Caspases—particularly Caspase-3—are key mediators of apoptosis in mammalian cells [65]. Caspase-3 activity was measured using Ac-DEVD-AMC, a fluorogenic substrate that contains a specific amino acid sequence selectively cleaved by Caspase-3. Upon cleavage, the substrate releases AMC, a fluorescent product that can be quantified using a spectrofluorometer.

**Figure 7:**
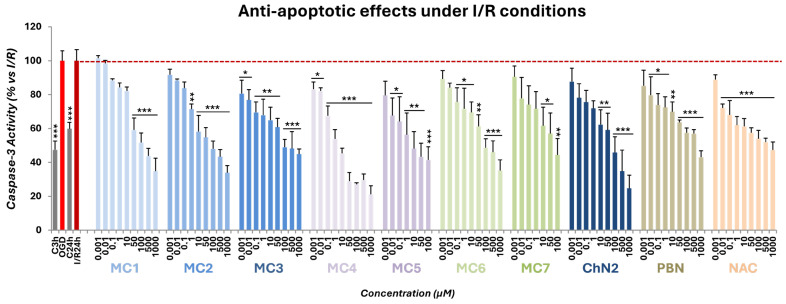
Anti-apoptotic effects of **MC1–7**, **ChN2**, **PBN**, and **NAC** in SH-SY5Y human neuroblastoma cells following OGD (3 h) and subsequent I/R (24 h) (dotted line). C denotes control cells maintained under standard conditions of oxygen and glucose. C3h corresponds to the control condition for OGD, and C24h corresponds to the control condition for I/R. The bars represent the % of caspase-3 activity under the indicated experimental conditions. Data are shown as means ± SEM of three independent experiments, each performed in triplicate. The statistical analysis (one-way ANOVA followed by Holm–Sidak post hoc test) reveals significant differences in caspase-3 activity compared to the I/R condition (* *p* < 0.05; ** *p* < 0.01; *** *p* < 0.001).

Caspase-3 activity levels after OGD and I/R exposure were set to 100%, and values from their respective controls were normalized accordingly (47.48 ± 5.09% for **C3h** and 59.90 ± 3.64% for **C24h**; mean ± SEM; n = 6) (Figure 7). Based on these data, the net apoptotic effects of OGD and I/R relative to their controls were 52.51 ± 5.48% and 40.10 ± 5.28%, respectively, indicating that I/R did not significantly attenuate the apoptotic effect induced by OGD (Δ = 12.42 ± 5.28%; ns, one-way ANOVA) (Figure 7).

All newly synthesized nitrones and oximes significantly reduced caspase-3 activity at concentrations of 50 μM and above. Notably, nitrone **MC3** and oxime **MC4** exhibited robust anti-apoptotic effects across all tested concentrations, outperforming the reference compounds (Figure 7).

**Figure 8:**
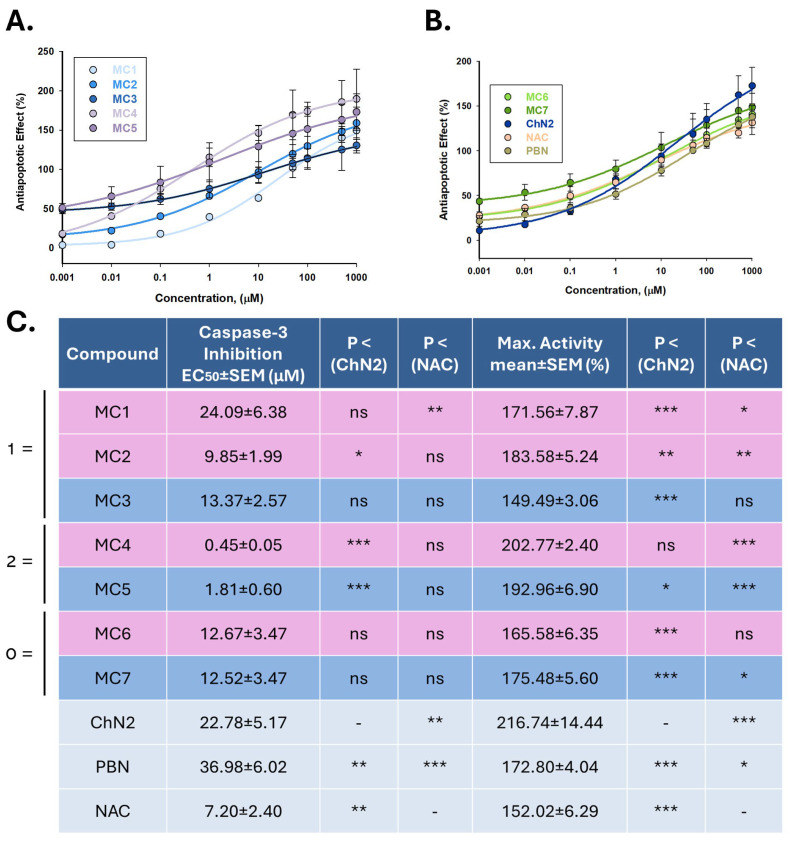
Anti-apoptotic effects of **MC1–7**, **ChN2**, **PBN**, and **NAC** in SH-SY5Y cells following I/R exposure. (**A**,**B**). Concentration–response curves showing the percentage of anti-apoptotic activity of each compound at the indicated concentrations. The curves were fitted using non-linear weighted regression analysis to estimate the effective dose 50 (EC_50_) and maximal anti-apoptotic activity (MAAA) values. Data represent the means ± SEM of three independent experiments, each performed in triplicate. (**C**) EC_50_ and MAAA values of the tested compounds. Rows highlighted in pink correspond to oximes, those in dark blue to nitrones, and the remaining to the three reference compounds. The structural labels 0 =, 1 =, and 2 = indicate the number of conjugated double bonds in the molecules: 0 = no double bonds; 1 = one double bond; 2 = two double bonds. Columns “*p* < (ChN2)” and “*p* < (NAC)” indicate the significance of the difference relative to **ChN2** and **NAC**, respectively. * *p* < 0.05, ** *p* < 0.01, and *** *p* < 0.001; ns: not statistically significant (one-way ANOVA with Holm–Sidak post hoc test).

Concentration–response curves for the anti-apoptotic effects of the test compounds were fitted using non-linear regression analyses to determine their potency and efficacy in preventing apoptosis (Figure 8). The EC_50_ values, from lowest to highest, were as follows: **MC4** ≤ **MC5** ≤ **NAC** ≤ **MC2** ≤ **MC7** ≤ **MC6** ≤ **MC3** ≤ **ChN2** ≤ **MC1** ≤ **PBN** (Figure 8C). Thus, several test compounds displayed significantly lower EC_50_ values than the reference cholesteronitrone **ChN2**, indicating higher anti-apoptotic potency. In terms of efficacy, the maximal anti-apoptotic activity (MAAA) values ranked as follows: **ChN2** ≥ **MC4** ≥ **MC5** ≥ **MC2** ≥ **MC7** ≥ **PBN** ≥ **MC1** ≥ **MC6** ≥ **NAC** ≥ **MC3** (Figure 8C). **ChN2** proved to be the most effective compound overall, whereas most of the newly synthesized compounds exhibited significantly higher MAAA values than **NAC**.

When comparing nitrones and oximes directly, EC_50_ analyses revealed no significant overall differences between both moieties, except within group 1, where nitrone **MC3** was more potent than oxime **MC1**, though not than oxime **MC2** (Figure S1C’, Supplementary Material). Across the three structural groups, mifepristone-derived compounds (group 2) consistently exhibited the lowest EC_50_ values (**MC4** < **MC2** ≤ **MC6** for oximes; **MC5** < **MC3** ≤ **MC7** for nitrones) and the highest MAAA values (**MC4** > **MC1** ≥ **MC6** for oximes; **MC5** > **MC1** ≥ **MC7** for nitrones) (Figure S1C’, Supplementary Material). These results suggest that the degree of conjugation in the molecular structure influences the anti-apoptotic properties of the compounds. Specifically, **MC4** and **MC5**, which contain two conjugated double bonds, demonstrated the most favorable profiles, outperforming the other oximes, nitrones, and reference compounds in reducing caspase-3 activity.

### 2.3. Antioxidant Profiles of NSNs, NSOs, ChN2, PBN and NAC

#### 2.3.1. Cell-Free Antioxidant Assays

The seven test compounds were assessed for their antioxidant and anti-inflammatory activities using a series of cell-free in vitro assays (Table 1).

##### Estimation of Lipophilicity (ClogP)

Lipophilicity, commonly expressed as the calculated logarithm of the partition coefficient (ClogP), is defined as the logarithm of the ratio between a compound’s concentration in a lipophilic phase (typically n-octanol) and in a hydrophilic phase (buffer 7.4) at equilibrium. This parameter provides critical insight into a compound’s ability to cross biological membranes, which strongly influences its biological activity. Herein, theoretical ClogP values were calculated using the Bioloom software (version 5.0) [66].

According to the calculated ClogP values (Table 1), comparison of ethisterone-derived (**MC1–3**) with stanolone-derived compounds (**MC6** and **MC7**) shows that the nitrones **MC3** and **MC7** are slightly more lipophilic than their corresponding oximes, with **MC7** being more lipophilic than **MC3**. In contrast, among the mifepristone-derived compounds (**MC4** and **MC5**), the oxime **MC4** is significantly more lipophilic than the nitrone **MC5**. It is also noteworthy that the mifepristone nitrone **MC5** is more lipophilic than the stanolone nitrone **MC7**.

##### Inhibition of Lipid Peroxidation (ILPO)

AAPH is a water-soluble azo compound frequently used as a thermal free radical generator in the study of inhibition of lipid peroxidation [67]. Upon decomposition, it produces molecular nitrogen and two carbon radicals, which can either combine to form stable products or react with molecular oxygen to generate peroxyl radicals [68]. These peroxyl radicals induce the lipid peroxidation of linoleic acid, leading to the formation of conjugated diene hydroperoxides [68].

All compounds showed very low ILPO values (2.4–19%), with oximes **MC4** and **MC6** exhibiting no inhibition under the experimental conditions (Table 1). Lipophilicity does not appear to play any significant role in this process.

##### DPPH Radical Scavenging Activity

The DPPH assay is a commonly used method to evaluate the ability of compounds to reduce the stable free radical 2,2-diphenyl-1-picrylhydrazyl (DPPH), which undergoes a color change upon reduction [66]. This interaction provides a measure of radical scavenging ability in an iron-free system. The underlying chemical reaction involves the reduction of DPPH radicals through electron transfer from the antioxidant. Particularly effective in this context are phenolic compounds, where phenoxide anions—such as those derived from catechol or its derivatives, including NDGA—act as strong electron donors.

As shown in Table 1, in this assay the test compounds exhibited low reducing activity (4.3–36%), with the highest values observed for **MC3** (12%), **MC7** (18.6%), and **MC6** (36%). This modest activity may be attributed, at least in part, to steric hindrance, as the bulky molecular scaffolds of these compounds could limit their accessibility to the DPPH radical’s reactive site [69].

##### Hydroxyl Radical Scavenging

The hydroxyl radical (•OH) is considered the most toxic ROS. It is directly involved in oxidative stress signaling and is a major contributor to oxidative damage to proteins, nucleic acids, and lipids [68]. In the central nervous system (CNS), polyunsaturated fatty acids are present at high concentrations and are particularly susceptible to free radical-mediated peroxidation. Furthermore, iron released from damaged brain cells can readily catalyze the generation of •OH radicals, exacerbating oxidative injury. To evaluate the hydroxyl radical scavenging activity of the synthesized oximes and nitrones, we used a competition assay with DMSO for hydroxyl radicals generated by the Fe^3+^/ascorbic acid system. The results were expressed as the % inhibition of formaldehyde production, which served as an indicator of radical scavenging capacity [43].

As shown in Table 1, all compounds displayed substantial activity (51.5–100%) compared to the reference antioxidant Trolox. Notably, the three most effective •OH scavengers were oximes [**MC1** (100%) > **MC4** (99%) > **MC6** (95%)], whereas among nitrones, **MC7** exhibited the lowest activity (51.5%). Among the three nitrones—**MC3**, **MC5**, and **MC7**—lipophilicity did not appear to correlate with the scavenging activity. In contrast, stereochemistry and functional group substitution seemed to influence activity. For instance, oximes **MC1** and **MC6**, where the nitrone group is replaced by a C=NOH moiety, were the most potent derivatives. Additionally, the presence of a carbon chain substituent with a triple bond seems to be associated with a slight enhancement in activity (**MC6**: 95%, **MC1**: 100%). Stereochemical configuration also played a role: **MC1**, with an E/Z ratio of 2:1, exhibited greater activity than **MC2** (E/Z = 4:1). Furthermore, a direct comparison between nitrone **MC7** and its oxime analog **MC6** revealed that the oxime displayed nearly twice the scavenging capacity. Overall, these findings suggest that higher lipophilicity tends to reduce •OH scavenging activity, whereas stereochemistry and oxime substitution enhance it.

##### ABTS•+ Decolorization Assay

The ABTS•+ decolorization assay is a widely used method for evaluating the antioxidant capacity of compounds based on their ability to scavenge the ABTS•+ radical cation. This radical is generated by the oxidation of ABTS with potassium persulfate and is characterized by a distinctive blue-green color. Upon addition of electron-donating antioxidants, ABTS•+ is reduced, resulting in a measurable decrease in absorbance [70].

In this assay, most of the test compounds displayed relatively low activity (4.5–40.5%). Among them, nitrone **MC7**, which contains a hydroxyl substituent, exhibited the highest antioxidant effect.

##### In Vitro Inhibition of Soybean LOX

Soybean lipoxygenases (LOXs) are enzymes that catalyze the formation of hydroperoxides from polyunsaturated fatty acids such as linoleic and arachidonic acid [71,72]. Upon stimulation, neutrophils cleave arachidonic acid from membrane phospholipids, leading to leukotriene production through the LOX pathway [71,72]. Leukotrienes are key mediators in several inflammatory diseases and are implicated in diverse pathophysiological processes, including skin disorders, cardiovascular disease, asthma and cancer [73,74].

We evaluated the new compounds as potential LOX inhibitors to assess their anti-inflammatory and antioxidant activities. Previous reports have suggested that LOX inhibition is related to lipophilicity [75], which is also supported by our findings. Analysis of the IC_50_ values (Table 1) identified oxime **MC4** as the most potent LOX inhibitor among the tested compounds (IC_50_ = 3.8 μM), followed by nitrone **MC7** (IC_50_ = 10.5 μM).

In conclusion, the results highlight the following: (a) the non-conjugated stanolone-derived nitrone **MC7**, with the second highest ClogP, was the most potent antioxidant in the ABTS•+ decolorization assay and ranked second in both the DPPH radical scavenging and ILPO assays; (b) the non-conjugated stanolone-derived oxime **MC6** was the most effective scavenger of DPPH radicals (36%); (c) the conjugated mifepristone-derived nitrone **MC5** showed the highest ILPO activity (19%); (d) the conjugated mifepristone-derived oxime **MC4**, together with the ethisterone-derived oxime **MC1**, was the most effective •OH scavenger (99%), as well as the most potent LOX inhibitor (IC_50_ = 3.8 µM); and (e) the conjugated ethisterone-derived nitrone **MC3** exhibited the second-highest LOX inhibitory activity (IC_50_ = 62 μM) and strong hydroxyl radical scavenging activity (74.2%), whereas its oxime counterpart **MC2** was the third most potent LOX inhibitor (IC_50_ = 97 µM). Despite these findings, establishing consistent structure–activity relationship (SAR) trends remains challenging. Nevertheless, non-conjugated nitrones such as **MC7** emerge as the most promising antioxidant candidates.

#### 2.3.2. Superoxide Radical Scavenging in a Cellular Model of Cerebral Ischemia

To evaluate the antioxidant properties of the test compounds, we assessed the rate of ROS production in SH-SY5Y cells following OGD (3 h) and subsequent reperfusion (2.5 h). Superoxide radical anion (O_2_•^−^) levels were quantified using dihydroethidium (DHE) as a fluorogenic probe [76].

**Figure 9:**
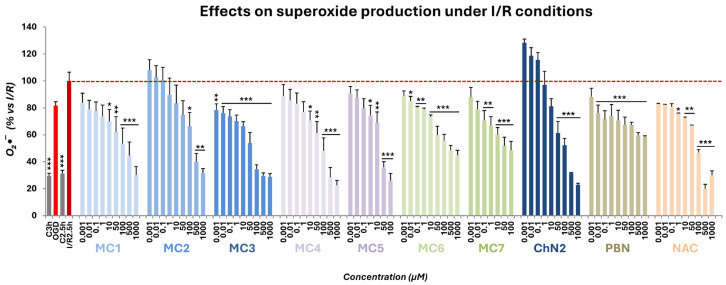
Effects of **MC1–7**, **ChN2**, **PBN**, and **NAC** on superoxide (O_2_•^−^) production in SH-SY5Y human neuroblastoma cells following OGD (3 h) and subsequent I/R (2.5 h) (dotted line). C denotes control cells maintained under standard conditions of oxygen and glucose. C3h corresponds to the control condition for OGD, and C2.5h corresponds to the control condition for I/R. The bars represent the % of superoxide anion (O_2_•^−^) generated under the indicated experimental conditions. Data are shown as means ± SEM of three independent experiments, each performed in triplicate. The statistical analysis (one-way ANOVA followed by Holm–Sidak post hoc test) reveals significant differences in O_2_•^−^ production compared to the I/R condition (* *p* < 0.05; ** *p* < 0.01; *** *p* < 0.001).

As shown in Figure 9, O_2_•^−^ production following I/R exposure was set to 100%, and values from OGD (81.55 ± 1.84%; mean ± SEM; n = 6) and their respective controls (29.64 ± 1.84% for C3h and 31.30 ± 2.42% for C2.5h; mean ± SEM; n = 6) were normalized accordingly. Based on these data, the net increases in O_2_•^−^ production relative to controls were 51.91 ± 2.37% for OGD and 68.71 ± 4.47% for I/R, indicating that reperfusion significantly enhanced superoxide generation compared with OGD alone (Δ = 16.80 ± 3.42%; *p* < 0.01, one-way ANOVA) (Figure 9).

The novel nitrones and oximes significantly reduced O_2_•^−^ production at intermediate and high concentrations, with **MC3** and **MC6** standing out due to their broad concentration range of activity. Overall, all seven novel compounds exhibited stronger antioxidant effects than **ChN2** and showed comparable efficacy to **NAC** (Figure 9).

**Figure 10:**
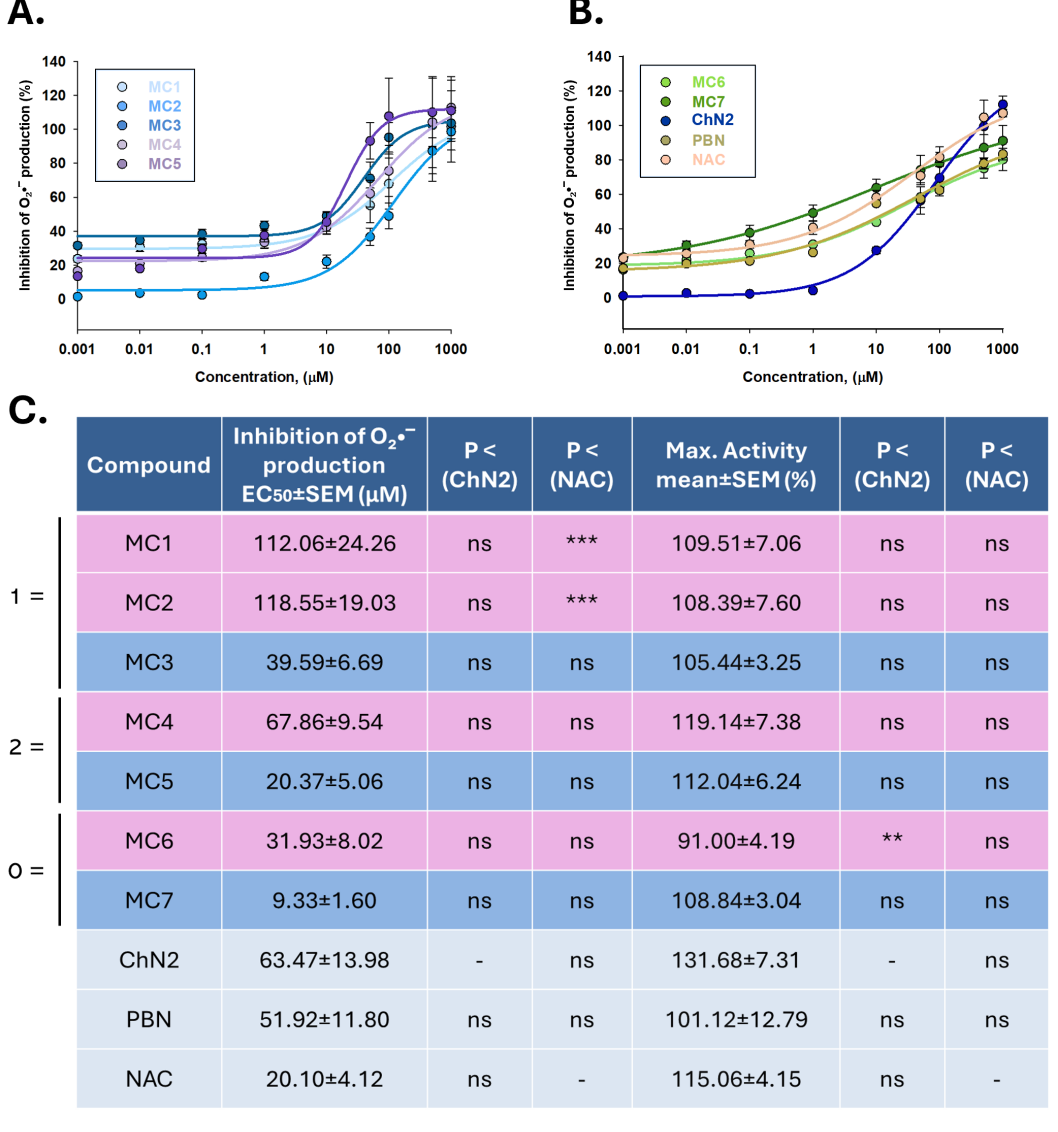
Inhibitory effects of **MC1–7**, **ChN2**, **PBN**, and **NAC** on superoxide (O_2_•^−^) production in SH-SY5Y cells following OGD (3 h) and subsequent reperfusion (2.5 h). (**A**,**B**) Concentration–response curves showing the percentage of antioxidant activity of each compound at the indicated concentrations. The curves were fitted using non-linear weighted regression analysis to estimate the effective dose 50 (EC_50_) and maximal antioxidant activity (MAOA) values. Data represent the means ± SEM of three independent experiments, each performed in triplicate. (**C**) EC_50_ and MAOA values of the tested compounds. Rows highlighted in pink correspond to oximes, those in dark blue to nitrones, and the remaining to the three reference compounds. The structural labels 0 =, 1 =, and 2 = indicate the number of conjugated double bonds in the molecules: 0 = no double bonds, 1 = one double bond, 2 = two double bonds. Columns “*p* < (ChN2)” and “*p* < (NAC)” indicate the significance of the difference relative to **ChN2** and **NAC**, respectively. ** *p* < 0.01, and *** *p* < 0.001; ns: not statistically significant (one-way ANOVA with Holm–Sidak post hoc test).

Concentration–response curves for the inhibition of O_2_•^−^ production, along with the EC_50_ values and maximal antioxidant activities (MAOAs) of the test compounds are presented in Figure 10. The order of EC_50_ values, from lowest to highest, was as follows: **MC7** ≤ **NAC** ≤ **MC5** ≤ **MC6** ≤ **MC3** ≤ **PBN** ≤ **ChN2** ≤ **MC4** < **MC1** ≤ **MC2** (Figure 10C). Thus, the potency of most of the newly developed compounds in reducing O_2_•^−^ generation was comparable to that of the reference compounds. In contrast, the two ethisterone-derived oximes (**MC1** and **MC2**) displayed significantly higher EC_50_ values than the rest of the compounds, including the ethisterone-based nitrone **MC3**. Conversely, the MAOA values followed this descending order: **ChN2** ≥ **NAC** ≥ **MC4** ≥ **MC5** ≥ **MC1** ≥ **MC7** ≥ **MC2** ≥ **MC3** ≥ **PBN** ≥ **MC6** (Figure 10C). Practically no statistically significant differences were observed between the MAOA values of the tested compounds and those of **ChN2** or **NAC**.

Regarding the comparison between nitrones and oximes, nitrones from all three structural groups (**MC3**, **MC5**, and **MC7**) were consistently more potent than their corresponding oximes (Figure S1D, Supplementary Material). Among them, nitrone **MC7**, which lacks conjugated double bonds, displayed the lowest EC_50_ and the highest potency in inhibiting O_2_•^−^ production (**MC7** > **MC5** ≥ **MC3**). In terms of efficacy, no statistically significant differences were observed between nitrones and oximes, except within group 3, where the MAOA of **MC7** was significantly higher than that of its oxime counterpart **MC6** (*p* < 0.05, one-way ANOVA) (Figure S1D’, Supplementary Material). Taken together, these results indicate that nitrones possess superior antioxidant properties compared with oximes, with nitrone **MC7** exhibiting the greatest antioxidant activity—comparable to **NAC** and higher than **ChN2**.

## 3. Discussion

Ischemia and reperfusion trigger cell death pathways that lead to neurological dysfunction [8,11,42]. Importantly, these processes are not entirely irreversible, which has fueled great interest in developing neuroprotective agents that, when administered in combination with current reperfusion strategies, may attenuate the activation of signaling cascades leading to cell death while enhancing cytoprotective responses [8,42].

Within this framework, the present study focused on the synthesis and in vitro evaluation of the neuroprotective and antioxidant effects of seven novel steroid-derived nitrones and oximes. Nitrones are well established spin-traps and free radical scavengers [22], whereas oximes are increasingly recognized for their anti-apoptotic, antioxidant, and anti-inflammatory properties [24,25,26]. By designing steroid-based nitrones and oximes, we sought to combine the functional activity of these moieties with the intrinsic lipophilicity of the steroidal backbone, thereby increasing the likelihood that the resulting molecules can cross the BBB and reach effective concentrations within the central nervous system. It is also worth noting that several neuroactive steroids themselves have shown considerable neuroprotective, anti-inflammatory, and neurogenic properties [32,35,36,37,38,39,41]. Surprisingly, despite being readily available molecules, the biological activity of such hybrid derivatives has received little attention, particularly in the context of ischemic stroke—with the exception of a few prior studies from our group [42,43,44].

Therefore, we set out to investigate the protective capacity of these seven newly synthesized steroid-derived compounds against two major forms of cell death involved in ischemia—necrosis and apoptosis—while also performing a detailed assessment of their antioxidant potential. For comparative purposes, all parameters were evaluated alongside cholesteronitrone **ChN2** [42,43], **PBN**, and **NAC**, which were used as standards. To this end, we employed cultures of the human neuroblastoma cell line SH-SY5Y as our experimental model. Although cell line-based systems do not fully reproduce the complexity of physiological conditions, they provide important advantages, including accessibility, ease of maintenance, a continuous supply of cells, and control over environmental variables, which enhances reproducibility [77,78]. Human in vitro models are also widely used in cost-effective high-throughput screening to evaluate therapeutic efficacy [77,78]. In particular, SH-SY5Y cells are a well-established model for studies on neuroprotection in ischemic stroke and neurodegenerative disorders [77,79,80].

Our results revealed that, among the seven newly synthesized steroid-derived compounds, nitrones **MC5** and **MC7** exhibited basal neurotoxic effects at high concentrations (500 and 1000 μM), similar to what was observed for **ChN2** in both the present study and previous reports [42,44] (Figure 2). However, most of the new test compounds displayed neuroprotective effects across a broad range of concentrations, reversing ischemia–reperfusion-induced loss of cell viability to a greater extent than **ChN2** and **PBN**, and in some cases comparably to **NAC** (Figure 3). Nitrones **MC3** (EC_50_ = 0.28 ± 0.12 μM) and **MC7** (EC_50_ = 0.08 ± 0.02 μM) were the most potent at low concentrations, whereas the mifepristone-derived nitrone **MC5** was significantly less potent than the rest of the novel compounds (Figure 4). Interestingly, with the exception of **MC5**, nitrones tended to be more potent than oximes in reversing the loss of metabolic activity in SH-SY5Y cells (Figure S1A). Conversely, oximes generally showed higher maximal neuroprotective activity (Figure S1A), with the ethisterone-derived oxime **MC2** standing out (MNA = 203.32 ± 5.83%) (Figure 4). This difference may be partly explained by the basal neurotoxic effects of nitrones at higher concentrations. Notably, all newly synthesized nitrones exhibited higher MNA values than **ChN2** and **PBN** (Figure 4). Regarding the influence of the degree of conjugation of the seven new molecules on their neuroprotective capacity, no significant differences were observed in potency; however, efficacy was affected, with ethisterone-derived compounds (containing one double bond in their structure) showing significantly higher efficacy than the other derivatives. Overall, all novel steroid-derived compounds show remarkable neuroprotective properties at low concentrations, which is of clinical relevance, as it suggests that lower doses of these compounds could be administered to patients to achieve therapeutic effects comparable to those of other agents, while potentially reducing the risk of adverse side effects.

The seven newly synthesized compounds exhibited strong anti-necrotic activity across a broad concentration range, achieving results comparable to those of the reference compounds (Figure 5). Among them, the stanolone-derived nitrone **MC7** emerged once again as the most potent agent (EC_50_ = 0.05 ± 0.02 μM), with nitrones in general showing stronger protective effects against necrosis than oximes at low concentrations (Figure S1B). By contrast, the mifepristone-derived compounds **MC4** and **MC5**—bearing two double bonds in their structure—displayed significantly higher EC_50_ values than the other nitrones and oximes, and were also less potent than **ChN2**, **PBN**, and **NAC** (Figure 6). Interestingly, the degree of molecular conjugation differentially influenced MANA values: the ethisterone-derived compounds—with a single double bond—were significantly less effective against necrosis compared to the others (Figure 6). Overall, the oxime **MC6** and the nitrone **MC7** displayed the most favorable anti-necrotic profiles, combining both potency and efficacy at levels comparable to or exceeding those of **ChN2** and **NAC** (Figure 6).

Regarding their anti-apoptotic capacity, compounds **MC1–7** exhibited a narrower range of effective concentrations, generally protecting SH-SY5Y cells from apoptosis at higher doses (Figure 7). Nevertheless, it is worth noting that all new compounds showed anti-apoptotic potency comparable to or greater than that of **ChN2** and **PBN** (Figure 8). In this case, no clear differences were observed between nitrones and oximes (Figure S1C,C’). However, the degree of conjugation played a decisive role in modulating the anti-apoptotic effect of the compounds, with the mifepristone-derived oxime **MC4** and nitrone **MC5** displaying significantly greater potency and efficacy than the rest (Figure 8).

This differential trend, whereby some compounds within the same set display stronger anti-necrotic effects while others predominantly exert anti-apoptotic activity, has already been reported in previous studies from our group [44]. In the present work, the stanolone-derived compounds **MC6** and **MC7** exhibited superior anti-necrotic effects, whereas the mifepristone-derived **MC4** and **MC5** showed more pronounced anti-apoptotic properties. These findings suggest that the steroidal scaffold and degree of conjugation critically shape the balance between anti-necrotic and anti-apoptotic activities. Consistent with our previous results, anti-necrotic mechanisms correlated more robustly with the overall neuroprotective capacity of the compounds than anti-apoptotic mechanisms [81]. Together with evidence showing that interventions targeting necrosis more consistently correlate with reductions in infarct volume and improvements in functional outcomes [82,83], this supports the preliminary selection of stanolone-derived compounds—particularly the nitrone **MC7**—as promising candidates for further in vivo evaluation in cerebral ischemia models.

In terms of the antioxidant activity of the new steroid-derived compounds, the main findings from the cell-free assays (Table 1) can be summarized as follows: (1) **MC1–7** exhibit lipophilicity comparable to or greater than that of NDGA and Trolox, which is a strong indicator of their ability to cross the BBB; (2) the mifepristone-derived oxime **MC4** (IC_50_ = 3.8 μM) and the stanolone-derived nitrone **MC7** (IC_50_ = 10.5 μM) were the most potent LOX inhibitors, indicating superior antioxidant and anti-inflammatory capacities compared with the other compounds [84]; (3) oximes generally showed greater hydroxyl radical scavenging activity than their corresponding nitrones, in many cases surpassing Trolox; and (4) nitrone **MC7** was the most effective ABTS·^+^ scavenger and the second most potent DPPH reducer, following oxime **MC6**. With respect to the ability of the compounds to reduce O_2_•^−^ production in SH-SY5Y cells exposed to the I/R model (Figure 9 and Figure 10), **MC7** emerged once again as the most potent compound (EC_50_ = 9.33 ± 1.60 μM). Furthermore, nitrones tended to exhibit greater potency in reducing O_2_•^−^ production than oximes, in line with their overall neuroprotective and anti-necrotic profiles (Figure S1D). However, no significant differences were observed among the MAOA values of **MC1–7** (Figure S1D’), which generally showed efficacy comparable to that of the reference compounds (Figure 10). Taken together, across all antioxidant assays performed, nitrone **MC7** stood out as the derivative with the most balanced and comprehensive antioxidant profile within the series of newly synthesized steroid-based molecules.

Finally, when linking the physiological roles and clinical uses of these nitrones and oximes with their neuroprotective capacity in cerebral ischemia, as investigated in this study, our findings indicate that nitrones structurally derived from stanolone (group 3, represented by **MC7**) are the most effective neuroprotectors, followed by nitrones derived from ethisterone (group 1, represented by **MC3**). This pattern is consistent with their known pharmacological properties: the former are potent androgenic metabolites of testosterone, clinically used to treat androgen deficiency [54], whereas the latter are synthetic progestogens historically employed to manage gynecological disorders [48]; both act as steroid receptor agonists. In contrast, the mifepristone-derived nitrones (group 2, represented by **MC5**) are steroid receptor antagonists with anti-progestogenic activity, clinically used for pregnancy termination [53].

Taken together, these results identify **MC7** as a hit neuroactive steroidal nitrone, exhibiting the most favorable neuroprotective, anti-necrotic, and antioxidant profile against ischemic injury. Based on these preliminary findings, **MC7** has been selected as a lead candidate for further characterization. Ongoing studies are currently aimed at elucidating its mechanisms of action in greater depth—including its potential modulation of inflammatory pathways, Nrf2 activation and other cell-death programs—before progression toward comprehensive in vivo evaluation in models of cerebral ischemia.

## 4. Materials and Methods

### 4.1. Chemistry

Please see the Supplementary Material.

### 4.2. Cell-Free Assays

Nordihydroguaiaretic acid (NDGA), Trolox, 2,2′-azobis(2-amidinopropane) dihydrochloride (AAPH), 2,2-diphenyl-1-picrylhydrazyl (DPPH), soybean lipoxygenase (LOX), ABTS, and linoleic acid sodium salt were purchased from Aldrich Chemical Co. (Milwaukee, WI, USA). A 0.1 M phosphate buffer (pH 7.4) was prepared by mixing 50 mL of 0.2 M KH_2_PO_4_ solution with 78 mL of 0.1 M NaOH solution, with the pH adjusted by the addition of KH_2_PO_4_ or NaOH as needed.

All in vitro assays were conducted using a PharmaSpec 1700 UV–Vis double-beam spectrophotometer (Shimadzu, Kyoto, Japan). Each experiment was performed in at least triplicate, and the standard deviation of absorbance values was less than 10% of the mean.

#### 4.2.1. Estimation of Lipophilicity (ClogP)

The calculated logarithm of the partition coefficient (ClogP), is defined as the logarithm of the ratio between a compound’s concentration in a lipophilic phase (typically octanol) and in a hydrophilic phase (typically water) at equilibrium. In general, higher ClogP values indicate greater lipophilicity, suggesting enhanced membrane permeability, although they may also imply an increased risk of non-specific binding and toxicity. Given its key role in shaping biological activity and Absorption–Distribution–Metabolism–Excretion–Toxicity (ADMET) properties, theoretical ClogP values were calculated using Bioloom software (version 5.0) from Biobyte Corp. (Available online: http://www.biobyte.com, accessed on 20 September 2025) (BioByte Corporation, C-QSAR database, 201 W Fourth St., Suite #204, Claremont, CA 91711-4707, USA).

#### 4.2.2. Determination of Reducing Activity (RA)

The DPPH assay is a commonly used method to evaluate the ability of compounds to reduce the stable free radical 2,2-diphenyl-1-picrylhydrazyl (DPPH), which undergoes a color change upon reduction. The test compounds were dissolved in DMSO to prepare a 10 mM stock solution. From this stock, 20 µL was added to 2 mL of a 0.05 mM ethanolic DPPH solution. The mixture was shaken vigorously and incubated at room temperature for 20 min. The absorbance at 517 nm was then recorded spectrophotometrically, and the percentage of reducing activity was calculated. Each assay was performed in three to four replicates, and the results were averaged. NDGA was used as a reference compound.

#### 4.2.3. Lipid Peroxidation Inhibition Assay

The inhibition of linoleic acid peroxidation was assessed using a UV–Vis spectrophotometric assay. In a UV cuvette, 930 µL of 0.05 M phosphate buffer (pH 7.4) was preincubated at 37 °C, followed by the addition of 10 µL of a 16 mM sodium linoleate solution. The oxidation reaction was initiated under aerobic conditions at 37 °C by adding 50 µL of a 40 mM AAPH solution, along with 10 µL of the test compound stock solution (10 mM in DMSO). Absorbance was recorded at 234 nm to monitor the oxidation rate, and results were compared to those obtained with Trolox as the reference compound.

#### 4.2.4. Hydroxyl Radical Scavenging Assay

The ability of the tested compounds to scavenge hydroxyl radicals was evaluated using a Fe^3+^/ascorbic acid system, with formaldehyde production from DMSO oxidation serving as the detection method. The reaction mixture contained EDTA (0.1 mM), Fe^3+^ (167 µM), DMSO (33 mM), and phosphate buffer (50 mM, pH 7.4), along with 10 µL of the test compounds stock solution (10 mM in DMSO) and 10 mM ascorbic acid. The reaction was incubated at 37 °C for 30 min in test tubes, after which trichloroacetic acid (TCA, 17% *w*/*v*) was added to stop the reaction. The percentage of hydroxyl radical scavenging activity was determined spectrophotometrically, with Trolox serving as a positive control.

#### 4.2.5. ABTS^•+^ Decolorization Assay

An ABTS stock solution (7 mM in water) was mixed with potassium persulfate (2.45 mM) and left in the dark at room temperature for 12–16 h to generate the ABTS•+ radical cation. For the assay, 10 µL of the test compounds stock solution (10 mM) was added to an ethanolic ABTS•+ solution and mixed thoroughly. Absorbance at 734 nm was measured 1 min after mixing to determine antioxidant activity. Trolox was used as the positive control.

#### 4.2.6. Soybean LOX Inhibition Assay

To evaluate the in vitro inhibition of soybean LOX, 10 µL of the test compound stock solution (10 mM in DMSO) was added to a UV cuvette at room temperature, along with 100 µL of sodium linoleate and 200 µL of enzyme solution (1.9 × 10^−4^ *w*/*v* in saline) prepared in Tris buffer (pH 9.0). The enzymatic conversion of sodium linoleate to 13-hydroperoxylinoleic acid was monitored by measuring absorbance at 234 nm. NDGA was used as the reference inhibitor. The % inhibition values reported in Table 1 of the Section 2 correspond to the effect measured at a single concentration (100 μM), whereas the IC_50_ values were derived from full concentration–response curves obtained using up to six different concentrations.

### 4.3. Cellular Model of Cerebral Ischemia

#### 4.3.1. Neuroblastoma Cell Cultures

SH-SY5Y human neuroblastoma cells (ATCC CRL-2266; LGC Limited, Luckenwalde, Germany) were maintained in culture flasks containing Dulbecco’s/Ham’s F12 medium (Gibco, Life Technologies, Madrid, Spain) supplemented with 2.5 mM GlutaMAX (Gibco, Life Technologies, Madrid, Spain), 1% antibiotic–antimycotic solution (100 mg/mL streptomycin, 100 µL/mL penicillin, and 0.25 mg amphotericin B) (Gibco, Life Technologies, Madrid, Spain), 1% gentamicin at a concentration of 15 mg/mL (Sigma-Aldrich, Madrid, Spain), and 10% fetal bovine serum (FBS) (Gibco, Life Technologies, Madrid, Spain), following standard protocols [44,81,85]. Cultures were incubated at 37 °C in a humidified atmosphere of 5% CO_2_ and 95% air. The culture medium was replaced every two days, and cells were passaged upon reaching confluence using 0.25% trypsin-EDTA (Gibco, Life Technologies, Madrid, Spain) for detachment and subsequent transfer into new flasks. For each experiment, SH-SY5Y cells were seeded into 48- or 96-well plates at densities of 1–1.5 × 10^5^ cells/well or 0.5 × 10^5^ cells/well, respectively, depending on the assay requirements.

#### 4.3.2. Exposure of Neuroblastoma Cell Cultures to Oxygen–Glucose Deprivation (OGD)

To model experimental ischemia (I), SH-SY5Y cells were subjected to oxygen–glucose deprivation (OGD) for 3 h. This was achieved by replacing the culture medium with glucose-free Dulbecco’s medium (Gibco, Life Technologies, Madrid, Spain) and incubating the plates in an anaerobic chamber maintained at 37 °C with a gas mixture of 95% N_2_ and 5% CO_2_ at 0.15 bar pressure [44,85]. Following the OGD period, the glucose-free medium was replaced with standard medium, and test compounds were added at concentrations ranging from 0.001 to 1000 µM. The cells were then incubated under normoxic conditions for 24 h to simulate reperfusion. While the term “ischemia–reperfusion” (I/R) is used herein, it is worth noting that “oxygen and glucose resupply” (OGD-R) is a more precise description for cellular models. Controls were conducted by maintaining SH-SY5Y cells in glucose-containing Dulbecco’s medium under normoxic conditions for 3 h, followed by a medium change and subsequent incubation under the same conditions for 24 h. Sodium nitroprusside (SNP) was included as a positive control for necrotic and apoptotic cell death at concentrations of 2 mM and 5 mM. Additionally, the vehicles used for compound dissolution (ethanol for **ChN2** and dimethyl sulfoxide for the rest of the molecules) were included at final concentrations of <2% in all experiments.

### 4.4. Neuroprotection Assays

#### 4.4.1. Evaluation of Cell Viability

Cell viability was assessed to determine the neurotoxic and neuroprotective effects of the tested compounds on SH-SY5Y human neuroblastoma cells seeded into 96-well culture plates at a density of 0.5 × 10^5^ cells/well. After exposure to either IR or control conditions, the compounds **MC1–7**, **ChN2**, **PBN**, and **NAC** were added at concentrations ranging from 0.001 to 1000 µM. Viability was then assessed using the Cell Proliferation Kit II (XTT) (ThermoFisher, Madrid, Spain), which measures cellular respiration by quantifying the reduction of yellow tetrazolium salt into an orange formazan dye by metabolically active cells [86]. Following the addition of the XTT solution (0.3 mg/mL), cells were incubated for 2 h at 37 °C in a humidified atmosphere containing 5% CO_2_ and 95% air. Absorbance was measured at 450 nm, with 650 nm used as a reference, using a Power-Wave XS microplate reader (BioTek Instruments, Madrid, Spain) [44,86]. A viability baseline of 100% was set by the control normoxic cells treated solely with standard Dulbecco’s medium. The effects of the test compounds were evaluated in three independent experiments, each performed in triplicate.

#### 4.4.2. Assessment of LDH Activity

The neuroprotective effects of the compounds against necrotic cell death were evaluated by measuring lactate dehydrogenase (LDH) levels in SH-SY5Y cells. Cells were seeded in 48-well plates at a density of 1–1.5 × 10^5^ cells/well and subjected to I/R conditions. **MC1–7**, **ChN2**, **PBN**, and **NAC** were added at concentrations ranging from 0.001 to 1000 µM. To measure extracellular LDH, the culture media from each well were collected and stored at −20 °C. For intracellular LDH, cells were treated with a lysis buffer containing 0.5% Triton X-100 in 0.1 M phosphate buffer (pH 7.5) to disrupt the membranes, followed by scraping the cells from the well bottoms. Both the collected supernatants (extracellular LDH) and the cell lysates (intracellular LDH) were subjected to the same subsequent steps. All samples were centrifuged at 13,000 rpm for 10 min to remove debris, and the resulting supernatants were analyzed. LDH activity was measured using a spectrophotometer (Power-Wave XS microplate reader, BioTek Instruments, Madrid, Spain) by monitoring the decline in absorbance at 340 nm, which corresponds to the oxidation of NADH to NAD+, as previously described [44,81]. Results were expressed as the ratio of extracellular LDH activity (indicative of necrotic cell death) to the total LDH activity (sum of extracellular and intracellular LDH) to normalize for cell density. The anti-necrotic effects of the test compounds were analyzed in three independent experiments, each performed in triplicate.

#### 4.4.3. Measurement of Caspase-3 Activity

The anti-apoptotic effects of the compounds were evaluated by assessing caspase-3 activity in SH-SY5Y human neuroblastoma cells. Cells were seeded in 48-well plates at a density of 1–1.5 × 10^5^ cells/well and subjected to I/R conditions. Following OGD exposure, **MC1–7**, **ChN2**, **PBN**, and **NAC** were added at concentrations ranging from 0.001 to 1000 µM, and the cells were incubated under normoxic conditions for a 24-h recovery period. Following incubation, the cells were lysed using a buffer containing 5 mM Tris-HCl (pH 8.0), 20 mM ethylenediaminetetraacetic acid (EDTA), and 0.5% Triton X-100. The resulting lysates were centrifuged at 13,000 rpm for 10 min to separate the cellular debris. Caspase-3 activity was determined by incubating the supernatants with the fluorogenic substrate N-Acetyl-Asp–Glu–Val–Asp-amido-4-methylcoumarin (Ac-DEVD-AMC) (Merck, Madrid, Spain), which releases a fluorescent product upon enzymatic cleavage. Fluorescence intensity was measured using a spectrofluorometer (Bio-Tek FL 600, BioTek Instruments, Madrid, Spain) with an excitation wavelength of 360 nm and an emission wavelength of 480 nm, following established protocols [44,81,86]. Prior to assessing caspase-3 activity, protein concentrations in the lysates were quantified using the Bradford assay to ensure standardized comparisons. The anti-apoptotic effects of the test compounds were evaluated across three independent experiments, each performed in triplicate.

### 4.5. Antioxidant Assays

#### Determination of Reactive Oxygen Species Formation

The antioxidant effects of the compounds were evaluated by measuring mitochondrial superoxide production in SH-SY5Y cells. Cells were seeded in 48-well plates at a density of 1–1.5 × 10^5^ cells/well and subjected to OGD for 3 h. Following OGD, the glucose-free medium was replaced with oxygenated Dulbecco’s medium, and the test compounds were added at concentrations ranging from 0.001 to 1000 µM. The plates were then incubated under normoxic conditions at 37 °C for 2.5 h. To detect superoxide production, the fluorogenic dye dihydroethidium (DHE, also known as hydroethidine) (Molecular Probes, ThermoFisher Scientific, Madrid, Spain) was added to the culture medium. Fluorescence measurements were recorded every 15–30 s over a 20-min period using a spectrofluorometer (Bio-Tek FL 600, BioTek Instruments, Madrid, Spain) with excitation and emission wavelengths of 535 nm and 635 nm, respectively [44,81]. The fluorescence data were analyzed using linear regression, and the resulting slopes (expressed in arbitrary fluorescence units, AFUs) were used as indicators of mitochondrial superoxide production, as described previously [44,81]. The antioxidant effects of the tested compounds were assessed in three independent experiments, with each experimental condition tested in triplicate.

### 4.6. Statistical Analyses

Data from cell culture experiments are presented as the means ± SEMs derived from three independent experiments using different cell batches, with each experimental condition tested in triplicate. Statistical comparisons between experimental groups were conducted using one-way analysis of variance (ANOVA), followed by the Holm–Sidak post hoc test, using GraphPad Prism version 8.0 (GraphPad Software, San Diego, CA, USA). A *p*-value of <0.05 was considered statistically significant. Fitting curves for effective dose 50 (EC_50_) and maximal activity determinations were generated with SigmaPlot v.11 (Systat Software INC., Palo Alto, CA, USA, 2012). EC_50_ values were estimated through non-linear regression analysis using logistic curve fitting, based on the equation: f1 = min + (max − min)/(1 + (x/EC50)^(−Hillslope)), where min and max represent the minimum and maximum responses, x is the compound concentration, and Hillslope defines the slope of the curve.

## 5. Conclusions

In this study, we conducted a comprehensive evaluation of the neuroprotective and antioxidant profiles of seven compounds (**MC1–7**)—three nitrones and four oximes derived from neuroactive steroids (NSNs and NSOs)—using an in vitro model of cerebral ischemia. Our findings demonstrate that, overall, the seven compounds exhibited superior protective capacities in this experimental model compared with two reference nitrones—cholesteronitrone **ChN2** and α-tert-butyl-nitrone (**PBN**)—as well as N-acetylcysteine (**NAC**). Moreover, NSNs displayed greater neuroprotective, anti-necrotic, and antioxidant potency than NSOs, regardless of the degree of conjugation in their molecular structures. Notably, the stanolone-derived nitrone **MC7**, which lacks conjugated double bonds, stood out among all tested compounds by exhibiting the most balanced and robust profile, consistently showing the highest potency in restoring cell viability, reducing necrotic cell death, and suppressing superoxide anion production. Consequently, **MC7** stands out as a promising lead compound, pending further mechanistic validation prior to its progression into in vivo studies of cerebral ischemia, where its capacity to reduce infarct volume and preserve neurological function will be further assessed.

## Figures and Tables

**Table 1:** In vitro antioxidant activities of neuroactive steroid–derived nitrones (NSNs) and oximes (NSOs). The assays included evaluated: inhibition of lipid peroxidation (ILPO), inhibition of soybean lipoxygenase (LOX), hydroxyl radical scavenging activity (•OH), scavenging activity of the cationic radical ABTS•^+^, and reducing activity based on interaction with the stable free radical DPPH.

Compounds ^a^/Standards ^a^	ClogP ^b^	ILPO (%)	LOX Inhibition (% or IC_50_) (μM)	^•^OH Scav. Activity (%)	ABTS^+●^ (%)	DPPH (%)
MC1 (O)	3.39	9.5 ± 0.7	12.0 ± 1.0%	100.0 ± 2.0	5.4 ± 0.1	4.3 ± 0.2
MC2 (O)	3.39	2.4 ± 0.3	97.0 ± 1.5 μM	57.0 ± 1.3	4.5 ± 0.3	4.8 ± 0.1
MC3 (N)	3.82	8.0 ± 0.1	62.0 ± 0.8 μM	74.2 ± 1.9	n.a.	12.0 ± 0.8
MC4 (O)	6.10	n.a.	3.8 ± 0.3 μM	99.0 ± 1.8	13.0 ± 0.4	7.8 ± 1.0
MC5 (N)	5.50	19 ± 1.1	n.a	77.3 ± 2.1	12.6 ± 0.6	11.0 ± 0.3
MC6 (O)	3.88	n.a	n.a	95.0 ± 2.7	9.0 ± 0.1	36.0 ± 1.2
MC7 (N)	4.06	13 ± 1.0	10.5 ± 0.7%	51.5 ± 1.7	40.5 ± 1.6	18.6 ± 0.7
NDGA	3.92	n.d.	0.5 ± 0.1%	n.d.	n.d.	96.0 ± 2.3
Trolox	3.09	93 ± 1.9	n.d.	88.0 ± 2.2	91.0 ± 2.0	n.d.

^a^ Compounds **MC1–7** were tested at a concentration of 100 µM. Data are shown as means ± SEM of three or four independent determinations. Means within each column differ significantly (*p* < 0.05). ^b^ BioByte Corporation, C-QSAR database, 201 W Fourth St., Suite #204, Claremont, CA 91711-4707, USA. n.a.: no activity detected under the experimental conditions; n.d.: not determined.

## Data Availability

The original contributions presented in the study are included in the article/Supplementary Materials, further inquiries can be directed to the corresponding author.

## Supplementary Materials

The following supporting information can be downloaded at: https://www.mdpi.com/article/10.3390/ijms262311506/s1.
