# Supplementary material for: Neuroprotective and Antioxidant Properties of Different Novel Steroid-Derived Nitrones and Oximes on Cerebral Ischemia In Vitro
Source: Int J Mol Sci. 2025 Nov 27;26(23):11506. doi: 10.3390/ijms262311506 (PMC12692713; doi:10.3390/ijms262311506)

Supplementary Material

Neuroprotective and Antioxidant Properties of Different Novel Steroid-Derived Nitrones and Oximes on Cerebral Ischemia in vitro

Sara Izquierdo-Bermejo [1,2,3,4], Mourad Chioua [5], Dimitra Hadjipavlou-Litina [6], Francisco López-Muñoz [3,7,8], José Marco-Contelles [5,9,*], and María Jesús Oset-Gasque [1,2,4,*]

[1] Department of Biochemistry and Molecular Biology, Faculty of Pharmacy, Complutense University of Madrid, Plaza Ramón y Cajal s/n, Ciudad Universitaria, 28040 Madrid, Spain
[2] Instituto de Investigación Sanitaria del Hospital Clínico San Carlos, 28040 Madrid, Spain
[3] Faculty of Health Sciences–HM Hospitals, Camilo José Cela University, Villafranca del Castillo, 28692 Madrid, Spain
[4] Instituto Universitario de Investigación en Neuroquímica, Complutense University of Madrid, Ciudad Universitaria, 28040 Madrid, Spain
[5] Laboratory of Medicinal Chemistry, Institute of Organic Chemistry (CSIC), C/Juan de la Cierva 3, 28006 Madrid, Spain
[6] Department of Pharmaceutical Chemistry, School of Pharmacy, Faculty of Health Sciences, Aristotle University of Thessaloniki, 54124 Thessaloniki, Greece.
[7] HM Hospitals Health Research Institute, 28015 Madrid, Spain
[8] Neuropsychopharmacology Unit, "Hospital 12 de Octubre" Research Institute, 28041 Madrid, Spain
[9] Center for Biomedical Network Research on Rare Diseases (CIBERER), Carlos III Health Institute (ISCIII), 28029 Madrid, Spain

* Correspondence: mjoset@ucm.es; jlmarco@iqog.csic.es

Content

Supplementary Figure 1 (S1) S2

General Methods.. S3

MC1, MC3... S4-S11

MC4, MC5... S12-S17

MC6, MC7... S18-S24

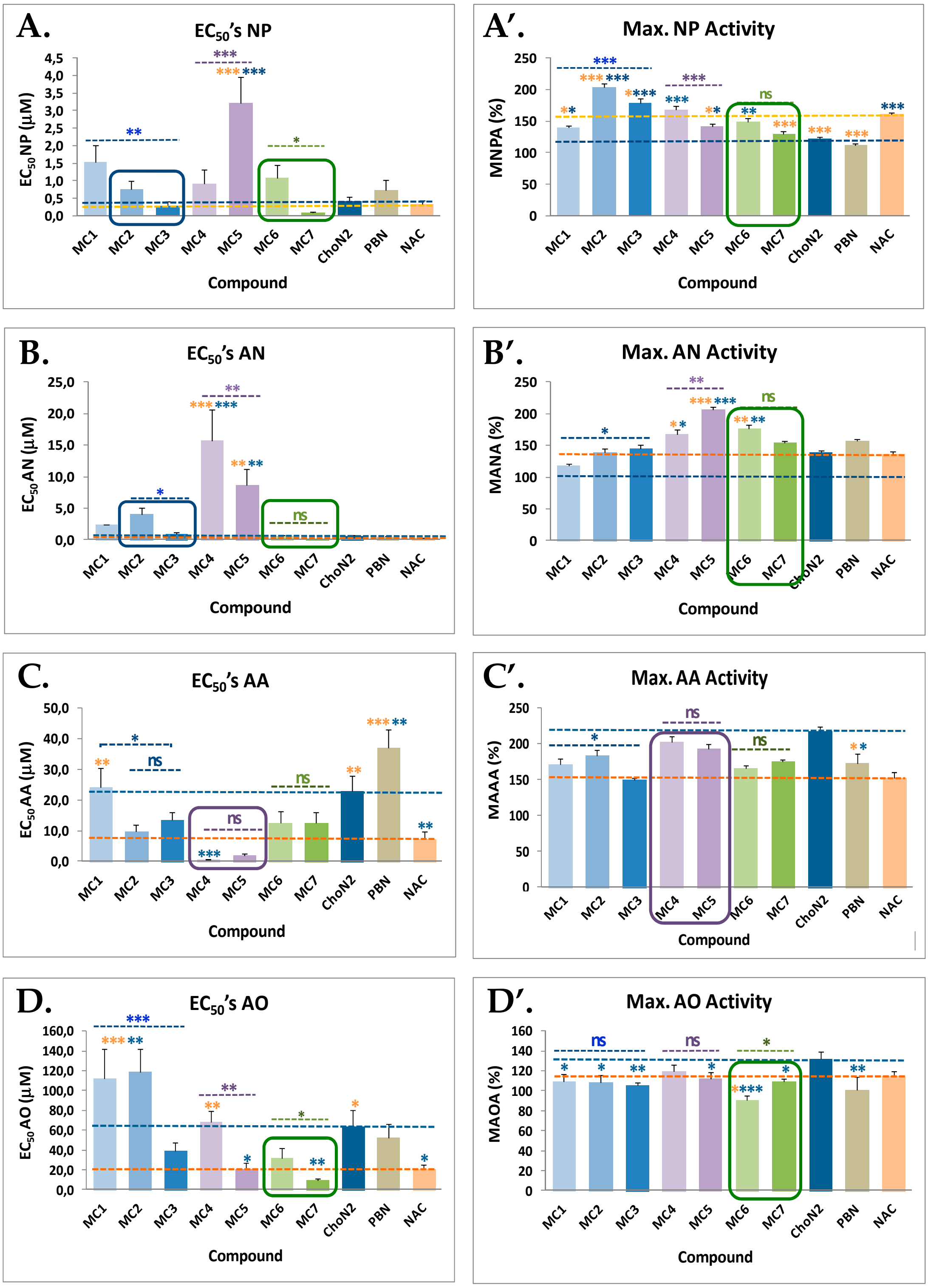

Figure S1. Statistical comparisons between EC$_{50}$ values (A–D) and maximal activities for neuroprotective (NP, A′), anti-necrotic (AN, B′), anti-apoptotic (AA, C′), and antioxidant (AO, D′) effects of the nitrones (MC3, MC5, MC7) and oximes (MC1, MC2, MC4, MC6) evaluated in this study. Statistical analyses of EC$_{50}$ and maximal activity values were performed using one-way ANOVA followed by the Holm–Sidak post hoc test (SigmaPlot v.11; Systat Software Inc., Palo Alto, CA, USA, 2012), by comparing everything against everything. The asterisks above the individual bars indicate statistical comparisons between nitrones and oximes with respect to NAC (orange asterisks) and **ChN2 (blue asterisks)**. Comparisons between nitrones

and oximes in each group are also indicated, in **light blue** for group derived from ethisterone, **purple** for group derived from mefipristone, and **green** for group derived from stanolone. ***p < 0.001, **p < 0.01, *p < 0.05. The squares indicate the most effective compounds for each activity.

Chemistry General Methods. Compound purification was performed by flash column chromatography on Silica Gel 60 (40–63 μm, Merck). Reactions were monitored using thin-layer chromatography (TLC) on Silica Gel 60 F_{254} (Merck) plates, and inspected under UV light (λ = 254 nm), and visualized with ethanolic solution of vanillin or ninhydrin. Melting points were determined using a Reichert Thermo Galen Kofler block and are uncorrected. ^{1}H NMR and ^{13}C NMR spectra were recorded on Bruker Avance 300 (300 MHz) and Bruker Avance 400 III HD (400 Hz) spectrometers. Samples were dissolved in $CDCl_3$ or DMSO-d_6 using TMS as internal standard for ^{1}H NMR spectra. In ^{13}C NMR spectra, $CDCl_3$ central signal (77.0 ppm) and DMSO-d_6 (39.5 ppm) were used as references. Chemical shifts (δ) are reported in *part per million* (ppm), and coupling constants (*J*) are quoted in Hz. Low resolution mass spectra were recorded on an Agilent HP 1100 LC/MS spectrometer, and High-Resolution Mass Specters (HRMS, Exact Mass) were measured on an AGILENT 6520 Accurate-Mass QTOF LC/MS spectrometer. Elemental analysis were recorded on a Carlo Erba EA 1108 apparatus, and confirmed to be ≥ 95%.

General Method for the Synthesis of the Oximes. A solution of the commercial ketone hydroxylamine hydrochloride (1.5 equiv) in pyridine (0.15 M) was heated at 90 °C until complete reaction (tlc control). Then, the solvent was evaporated and the crude mixture was purified on column chromatography to yield the oxime.

General Method for the Synthesis of the nitrones. A solution of the commercial ketone, Na_2SO_4 (3 equiv), AcONa (2 equiv) and *N*-methylhydroxylamine hydrochloride (1.5 equiv) in EtOH (0.15 M) was heated at 90 °C during 16 h. Then, the solvent was evaporated and the crude mixture was purified on column chromatography (CH_2Cl_2/MeOH, 0%-50%) to yield the nitrone.

MC1, MC3

MC1

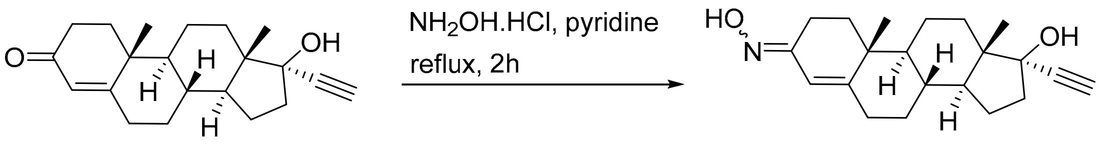

(8*R*,9*S*,10*R*,13*S*,14*S*,17*R*,*E*)-17-Ethynyl-17-hydroxy-10,13-dimethyl-1,2,6,7,8,9,10,11,12,13,14,15,16,17-tetradecahydro-3H-cyclopenta[a]phenanthren-3-one oxime (MC1) [Chopra, G. Compounds for targeted therapies of castration resistant prostate cancer. PCT Int. Appl. (**2020**), WO 2020176843; Matlin, S. A.; Jiang, L.; Roshdy, S.; Zhou, R. Resolution and identification of steroid oxime syn and anti isomers by HPLC. *J. Liq. Chromatogr.* **1990**, *13*, 3455-63]. Following the **General Method for the Synthesis of the Oximes**, a solution of commercial ethisterone {(8*R*,9*S*,10*R*,13*S*,14*S*,17*R*)-17-ethynyl-17-hydroxy-10,13-dimethyl-1,2,6,7,8,9,10,11,12,13,14,15,16,17-tetradecahydro-3*H*-cyclopenta[*a*]phenanthren-3-one} (624 mg, 2 mmol), hydroxylamine hydrochloride (207 mg, 3 mmol, 1.5 equiv) in pyridine (5 mL) was heated at 90 °C for 2 h. Then, the solvent was evaporated and the crude mixture was purified on column chromatography (hexane/AcOEt; 3/2) to yield compound **MC1** (white solid, 180 mg, 55%), as an unseparable mixture of isomers in a 2/1 ratio: mp > 230 ºC; ^1H NMR (300 MHz, DMSO-d_6) δ 10.45 [s, 1H, =N-OH (major isomer, MI)], 10.19 [s, 1H, =N-OH (minor isomer, mi)], 6.31 [s, 1H, H4 (mi)], 5.68 [s, 1H, H4 (MI)], 5.28 [s, 1H, C(17)OH], 3.28 (s, 1H, C≡CH), 2.89 (d, *J*= 16.0 Hz, 1H, H6, MI), 2.43-1.95 (m, 5H), 1.87-1.62 (m, 4H), 1.56-1.52 (m, 4H), 1.45-1.12 (m, 3H), 0.99-0.81 (m, 3H), 0.88 (s, 3H, CH_3), 0.78 (s, 3H, CH_3); MS (EI) *m*/*z*: 327 [M, 100]$^{+\cdot}$, 310 [M-OH, 63]$^{+\cdot}$, 292 [M-NOH, 48]$^{+}$. HRMS (ESI_ACN): Calcd. for [$C_{21}H_{29}NO_2$]$^+$: 328,2271.

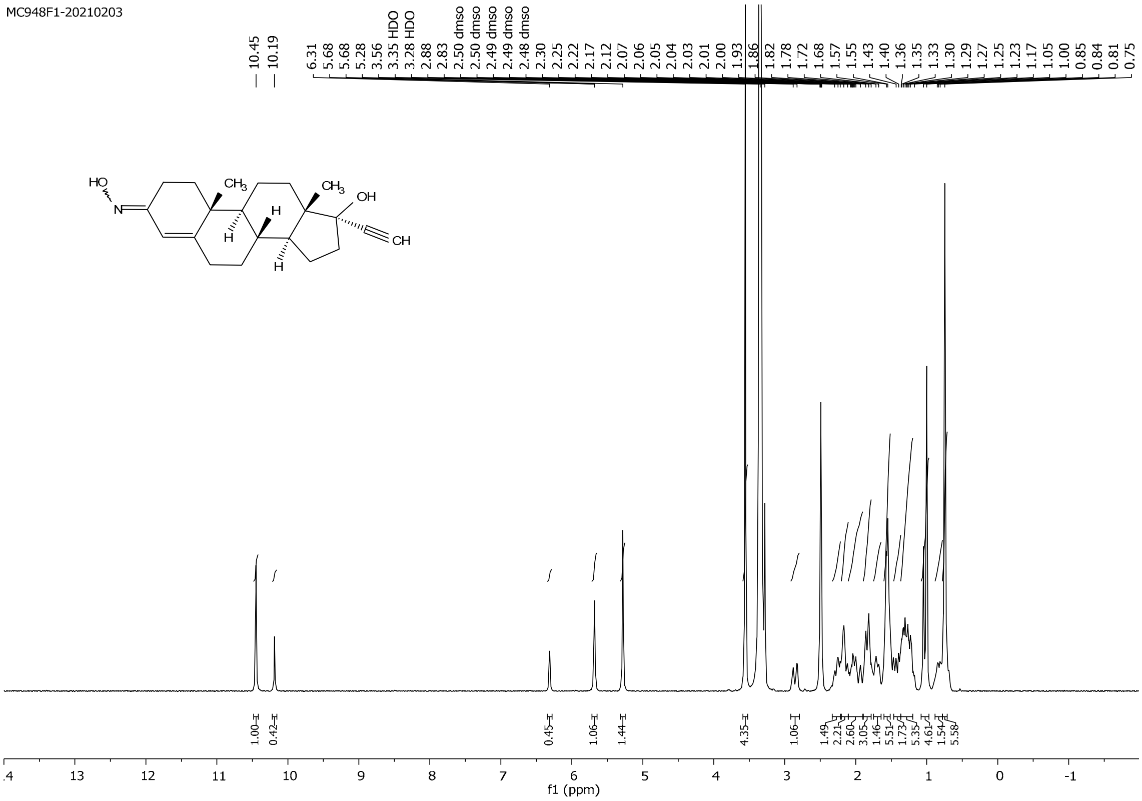

MC948F1-20210203
10.45
10.19
6.31
5.68
5.68
5.28
3.56
3.35 HDO
3.28 HDO
2.88
2.83
2.50 dmso
2.50 dmso
2.49 dmso
2.49 dmso
2.48 dmso
2.30
2.25
2.22
2.17
2.12
2.07
2.06
2.05
2.04
2.03
2.01
2.00
1.93
1.86
1.82
1.78
1.72
1.68
1.57
1.55
1.43
1.40
1.36
1.35
1.33
1.30
1.29
1.27
1.25
1.23
1.17
1.05
1.00
0.85
0.84
0.81
0.75
HO
N
CH3
CH3
OH
H
H
H
CH
1.00
0.42
0.45
1.06
1.44
4.35
1.06
1.49
2.21
2.60
3.05
1.46
5.51
1.73
5.35
4.61
1.54
5.58
f1 (ppm)

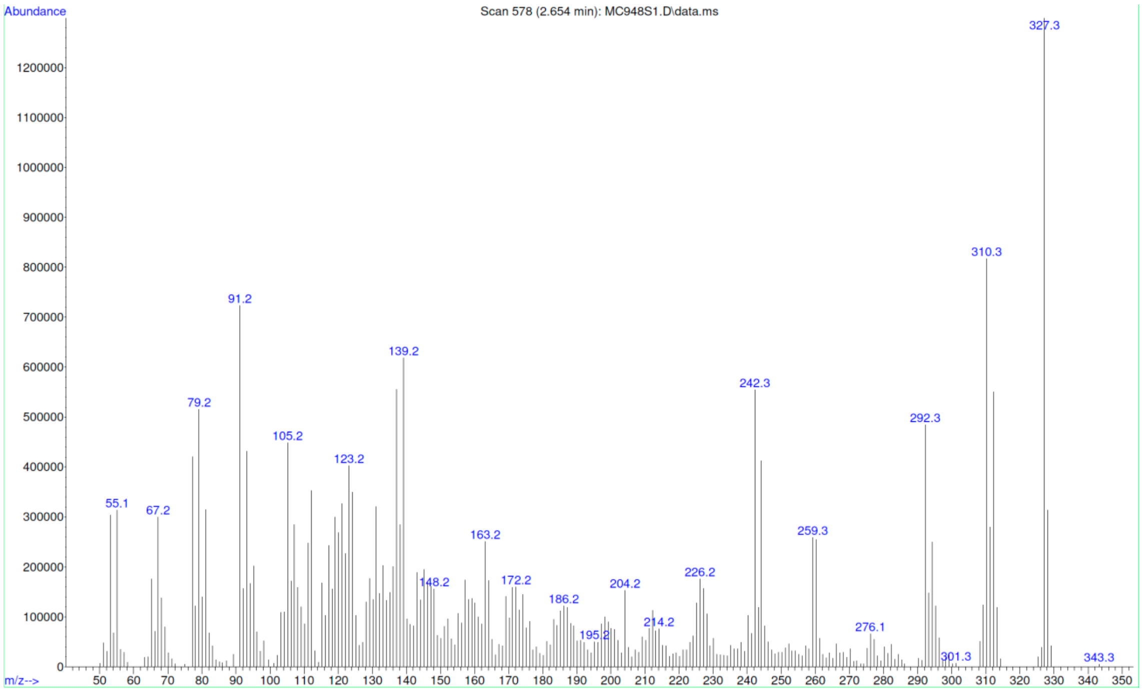

Abundance
Scan 578 (2.654 min): MC948S1.D\data.ms
327.3
310.3
91.2
139.2
242.3
79.2
292.3
105.2
123.2
55.1
67.2
163.2
259.3
226.2
148.2
172.2
204.2
186.2
214.2
195.2
276.1
301.3
343.3
m/z-->

**Data File**	209_MC948bS1_01.d	**Sample Name**	MC948bS1
**Sample Type**	Sample	**Position**	Vial 10
**Instrument Name**	Instrument 1	**User Name**	
**Acq Method**	ESI_ACN_75_pos_new.m	**Acquired Time**	2/10/2021 11:55:09 AM (UTC+01:00)
**IRM Calibration Status**	Success	**DA Method**	Defecto_modificado.m
**Comment**			

**Sample Group**		**Info.**	
**User**	MOURAD CHIOUA	**Stream Name**	LC 1
**Acquisition Time (Local)**	2/10/2021 11:55:09 AM (UTC+01:00)	**Acquisition SW Version**	6200 series TOF/6500 series Q-TOF B.08.00 (B8058.3 SP1)
**QTOF Driver Version**	8.00.00	**QTOF Firmware Version**	2.712
**Tune Mass Range Max.**	1700		

Compound Table

Compound Label	RT	Mass	Abund	Formula	Tgt Mass	Diff (ppm)	Hits (DB)
Cpd 1: C21 H29 N O2; 2.282	2.282	327.2188	293589	C21 H29 N O2	327.2198	-3.14	1

Compound Label	*m/z*	RT	Algorithm	Mass
Cpd 1: C21 H29 N O2; 2.282	328.2262	2.282	Find by Formula	327.2188

MS Zoomed Spectrum

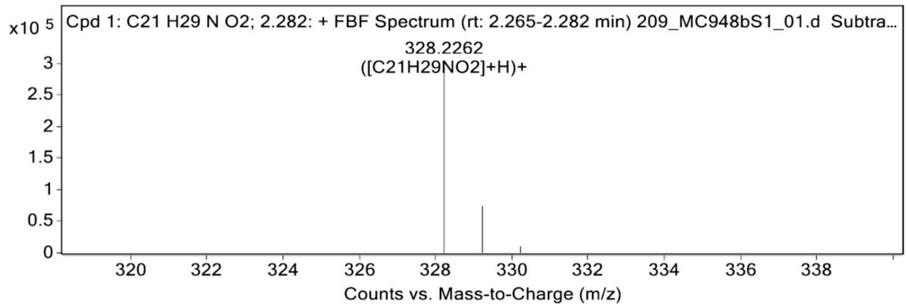

MS Spectrum Peak List

m/z	z	Abund	Formula	Ion
328.2262	1	293589.38	C21H29NO2	(M+H)+
329.2289	1	74119.82	C21H29NO2	(M+H)+
330.2321	1	10457.37	C21H29NO2	(M+H)+

MC3

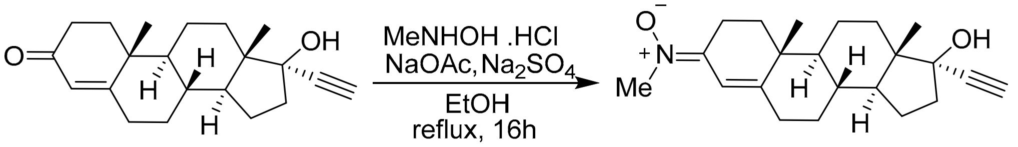

(8*R*,9*S*,10*R*,13*S*,14*S*,17*R*,*E*)-17-Ethynyl-17-hydroxy-*N*,10,13-trimethyl-1,2,6,7,8,9,10,11,12,13,14,15,16,17-tetradecahydro-3*H*-cyclopenta[*a*]phenanthren-3-imine oxide (MC3) [Weintraub, P. M.; Tiernan, P. L. Steroidal nitrones. *J. Org. Chem.* **1974**, *39*, 1061-5]. Following the **General Method for the Synthesis of the Nitrones**, a solution of commercial ethisterone {(8*R*,9*S*,10*R*,13*S*,14*S*,17*R*)-17-ethynyl-17-hydroxy-10,13-dimethyl-1,2,6,7,8,9,10,11,12,13,14,15,16,17-tetradecahydro-3*H*-cyclopenta[*a*]phenanthren-3-one} (312 mg, 1 mmol), Na_2SO_4 (426 mg, 3 mmol, 3 equiv), AcONa (164 mg, 2 mmol, 2 equiv) and *N*-methylhydroxylamine hydrochloride (125 mg, 1.5 mmol, 1.5 equiv) in EtOH (7 mL) was heated at 90 °C for 16 h. After that time, the solvent was evaporated and the crude mixture was purified on column chromatography (DCM/methanol 0%-50%) to yield compound **MC3** (white solid, 180 mg, 53%), isolated as an unseparable mixture of *E*/*Z* isomers in a 14/1 ratio (HPLC-MS analysis), as confirmed by analysis of the ^{1}H NMR nOe effect: mp > 230 ºC; ^{1}H NMR (400 MHz, DMSO-d_6) δ 6.18 (s, 1H), 5.27 (s, 1H), 3.55 (d, *J* = 1.7 Hz, 4H), 3.27 (s, 2H), 2.93 (dt, *J* = 16.8, 3.8 Hz, 1H), 2.33 – 2.22 (m, 2H), 2.22 – 2.16 (m, 1H), 2.06 (t, *J* = 9.6 Hz, 2H), 2.00 (dd, *J* = 9.1, 5.1 Hz, 1H), 1.81 (tdd, *J* = 16.9, 8.1, 3.0 Hz, 3H), 1.69 (dd, *J* = 12.1, 3.4 Hz, 1H), 1.53 (q, *J* = 9.8, 7.2 Hz, 5H), 1.49 – 1.37 (m, 2H), 1.33 (dt, *J* = 11.0, 5.8 Hz, 2H), 1.29 – 1.20 (m, 3H), 1.19 (s, 1H), 0.95 (s, 4H), 0.82 (tt, *J* = 12.5, 6.5 Hz, 2H), 0.72 (s, 4H), 0.70 – 0.65 (m, 1H); MS (EI) *m*/*z*: 341 [M, 100]$^{+\cdot}$, 325 [M-O, 33]$^{+\cdot}$, 310 [M-OCH_3, 20]$^+$. HRMS (ESI_ACN): Calcd. for $[C_{22}H_{31}NO_2]^+$: 342,2428. Found 342,2427 [M + 1]$^+$.

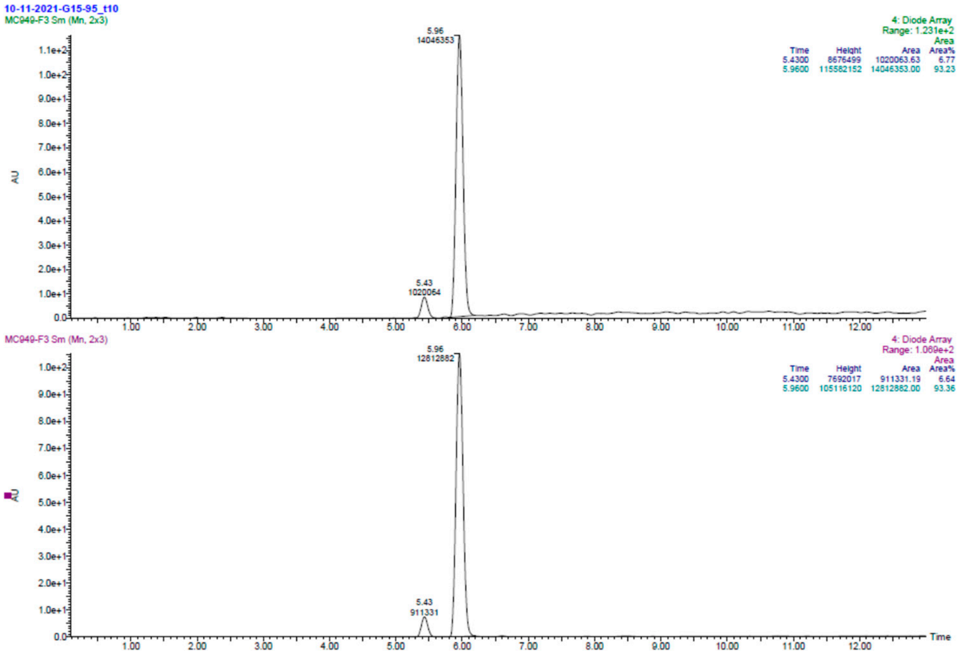

MCH_949F3

G15-95_10min

PDA 190-700nm
% área

PDA filtrado
240-400nm
% área

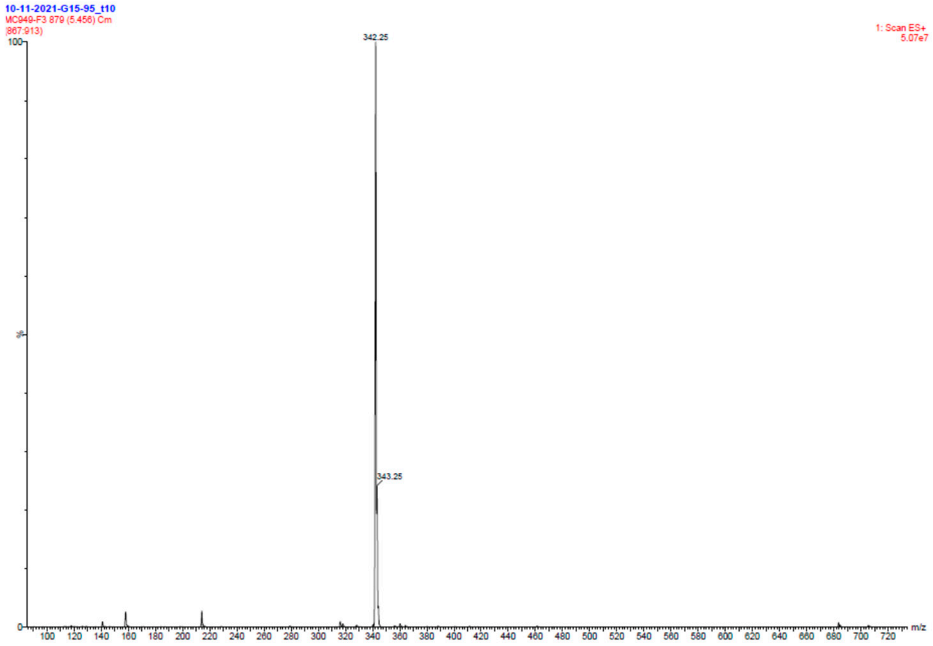

MCH_949F3

G15-95_10min

TIC 20V

Promedio de masas
en RT 5,46

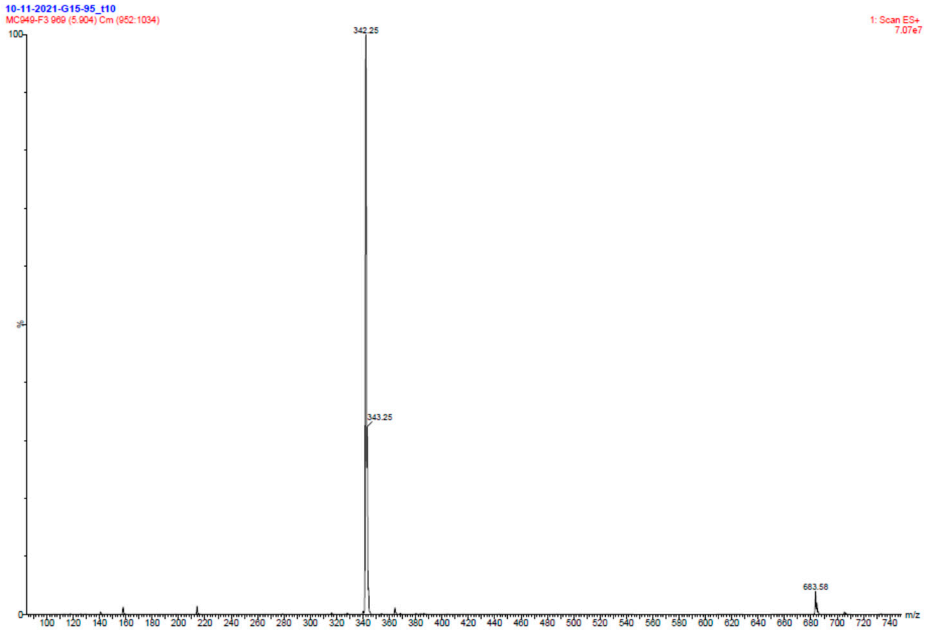

MCH_949F3
G15-95_10min
TIC 20V
Promedio de masas
en RT 5,90

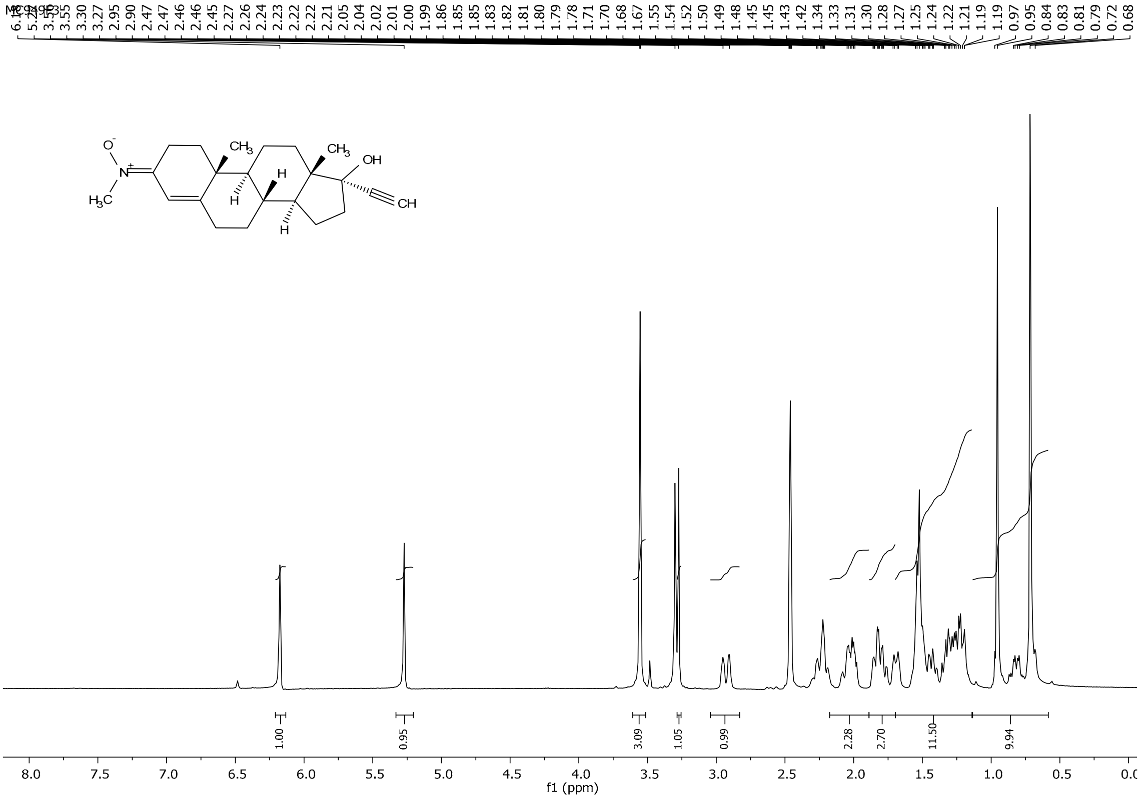

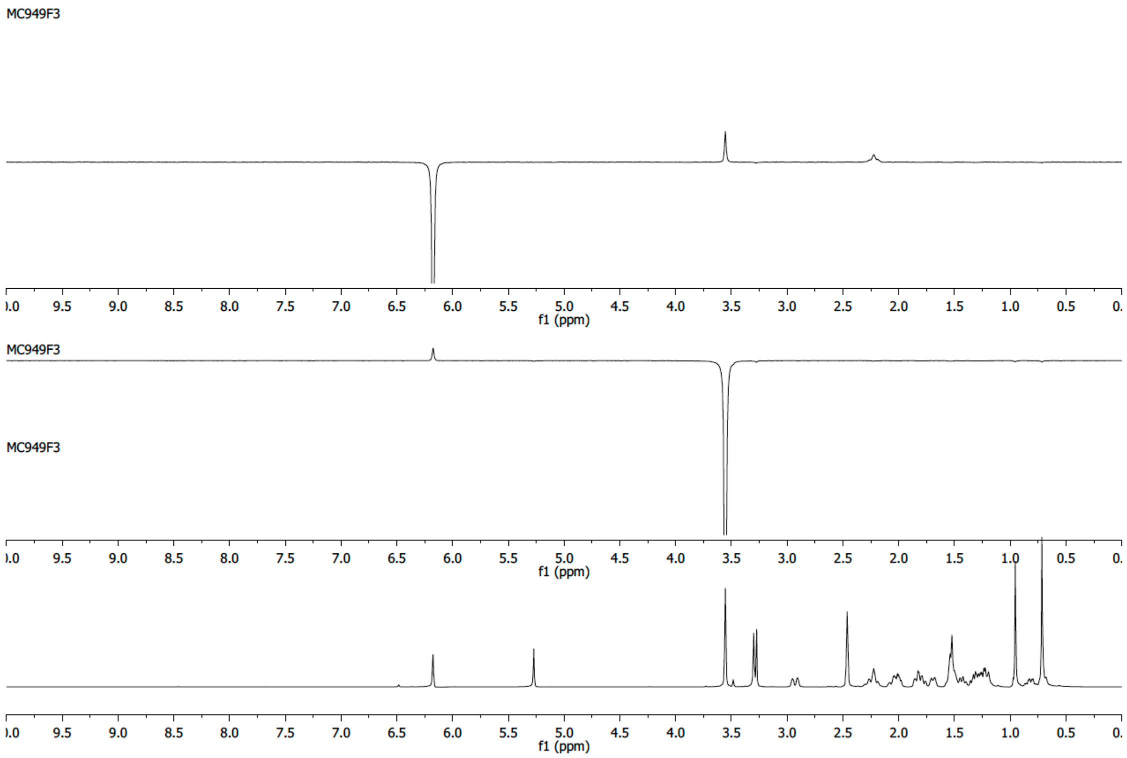
MC949F3
f1 (ppm)
MC949F3
MC949F3
f1 (ppm)
f1 (ppm)

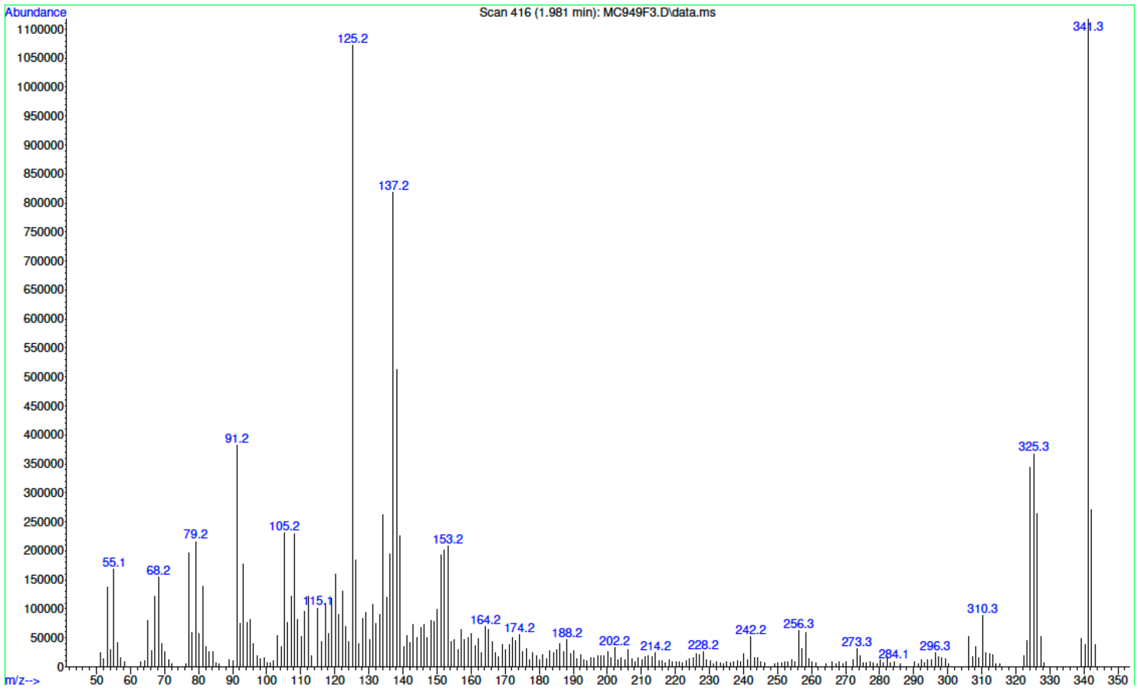
Abundance
Scan 416 (1.981 min): MC949F3.D\data.ms
m/z-->
125.2
137.2
91.2
325.3
341.3
105.2
153.2
79.2
55.1
68.2
115.1
164.2
174.2
188.2
202.2
214.2
228.2
242.2
256.3
273.3
284.1
296.3
310.3

**Data File**	192_MC949F3_01.d	**Sample Name**	MC949F3
**Sample Type**	Sample	**Position**	Vial 9
**Instrument Name**	Instrument 1	**User Name**	
**Acq Method**	ESI_ACN_75_pos_new.m	**Acquired Time**	2/8/2021 1:11:52 PM (UTC+01:00)
**IRM Calibration Status**	Success	**DA Method**	Defecto_modificado.m
**Comment**			

**Sample Group**		**Info.**	
**User**	MOURAD CHIOUA	**Stream Name**	LC 1
**Acquisition Time (Local)**	2/8/2021 1:11:52 PM (UTC+01:00)	**Acquisition SW Version**	6200 series TOF/6500 series Q-TOF B.08.00 (B8058.3 SP1)
**QTOF Driver Version**	8.00.00	**QTOF Firmware Version**	2.712
**Tune Mass Range Max.**	1700		

Compound Table

Compound Label	RT	Mass	Abund	Formula	Tgt Mass	Diff (ppm)	Hits (DB)
Cpd 1: C22 H31 N O2; 2.065	2.065	341.2352	988785	C22 H31 N O2	341.2355	-0.9	1

Compound Label	*m/z*	RT	Algorithm	Mass
Cpd 1: C22 H31 N O2; 2.065	342.2427	2.065	Find by Formula	341.2352

MS Zoomed Spectrum

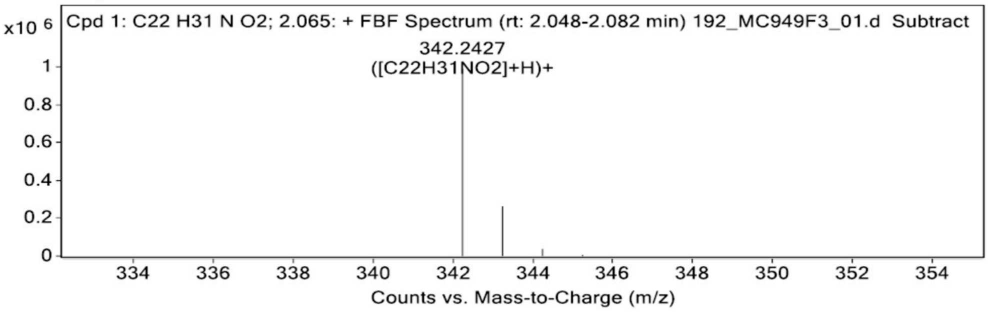

MS Spectrum Peak List

m/z	z	Abund	Formula	Ion
342.2427	1	988785.25	C22H31NO2	(M+H)+
343.245	1	262128.83	C22H31NO2	(M+H)+
344.2481	1	37091.6	C22H31NO2	(M+H)+
345.2474	1	4900.19	C22H31NO2	(M+H)+

MC4, MC5

MC4

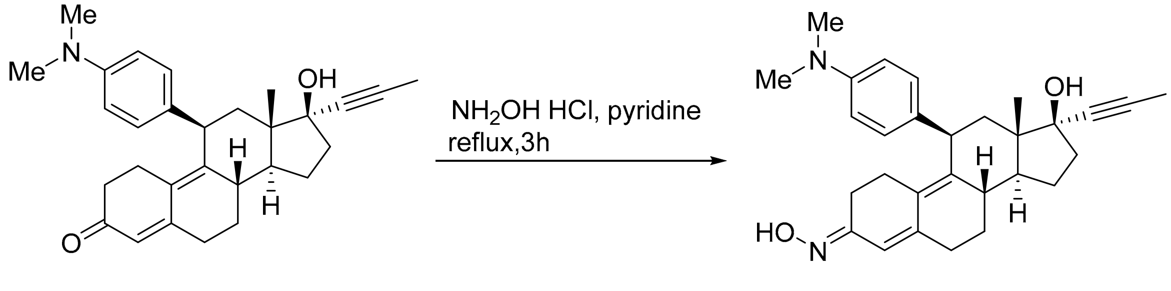

(8*S*,11*R*,13*S*,14*S*,17*S*,*E*)-11-(4-(Dimethylamino)phenyl)-17-hydroxy-13-methyl-17-(prop-1-yn-1-yl)-1,2,6,7,8,11,12,13,14,15,16,17-dodecahydro-3*H*-cyclopenta[α]phenanthren-3-one oxime *E*/*Z* : 3/2 (MC4) [Torelli, V.; Teutsch, J. G.; Phillbert, D. Steroid derivatives U.S. (1987) 4634695]. Following the **General Method for the Synthesis of the Oximes**, a solution of commercial mifepristone {(8*S*,11*R*,13*S*,14*S*,17*S*)-11-(4-(dimethylamino)phenyl)-17-hydroxy-13-methyl-17-(prop-1-yn-1-yl)-1,2,6,7,8,11,12,13,14,15,16,17-dodecahydro-3*H*-cyclopenta[*a*]phenanthren-3-one} (215 mg, 0.5 mmol), hydroxylamine hydrochloride (52 mg, 0.75 mmol, 1.5 equiv) in pyridine (5 mL) was heated at 90 °C during 3 h. Then, the solvent was evaporated and the crude mixture was purified on column chromatography (DCM/methanol 0%-10%) to yield compound **MC4** (white solid, 110 mg, 50%) as an unseparable mixture of isomers, in a 3/2 ratio: mp 180-2 ºC; ^{1}H NMR (300 MHz, $CDCl_3$) δ 7.04 (dd, *J* = 8.5, 5.9 Hz, 4H), 6.66 (dd, *J* = 8.8, 3.1 Hz, 4H), 6.51 (s, 1H), 5.86 (s, 1H), 4.30 (s, 2H), 2.91 (s, 11H), 2.68 – 2.53 (m, 3H), 2.53 – 2.44 (m, 3H), 2.44 – 2.14 (m, 10H), 2.09 – 1.86 (m, 11H), 1.72 – 1.59 (m, 3H), 1.47 – 1.20 (m, 5H), 0.53 (d, *J* = 2.2 Hz, 5H); MS (EI) *m*/*z*: 444 [M, 100]$^{+\cdot}$, 427 [M-OH, 12]$^+$. HRMS (ESI_ACN): Calcd. for $[C_{29}H_{36}N_2O_2]^+$: 445,2850. Found 445,2845 [M + 1]$^+$.

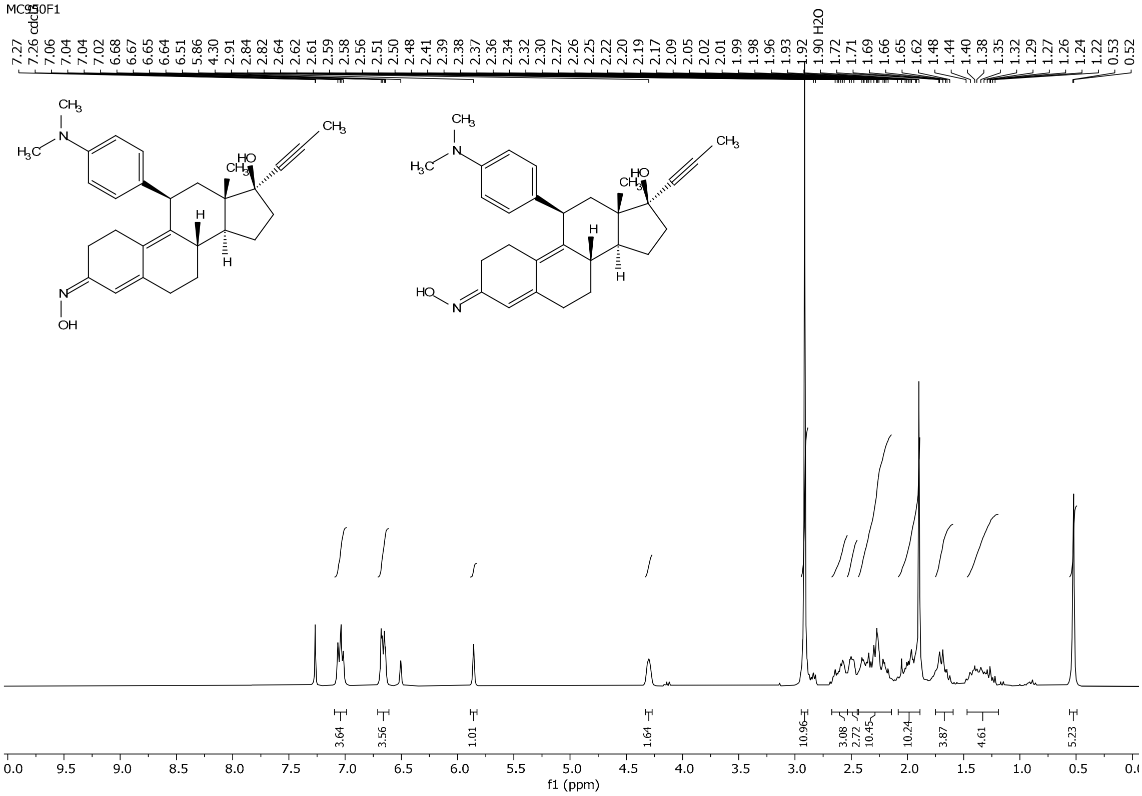
MC950F1
f1 (ppm)

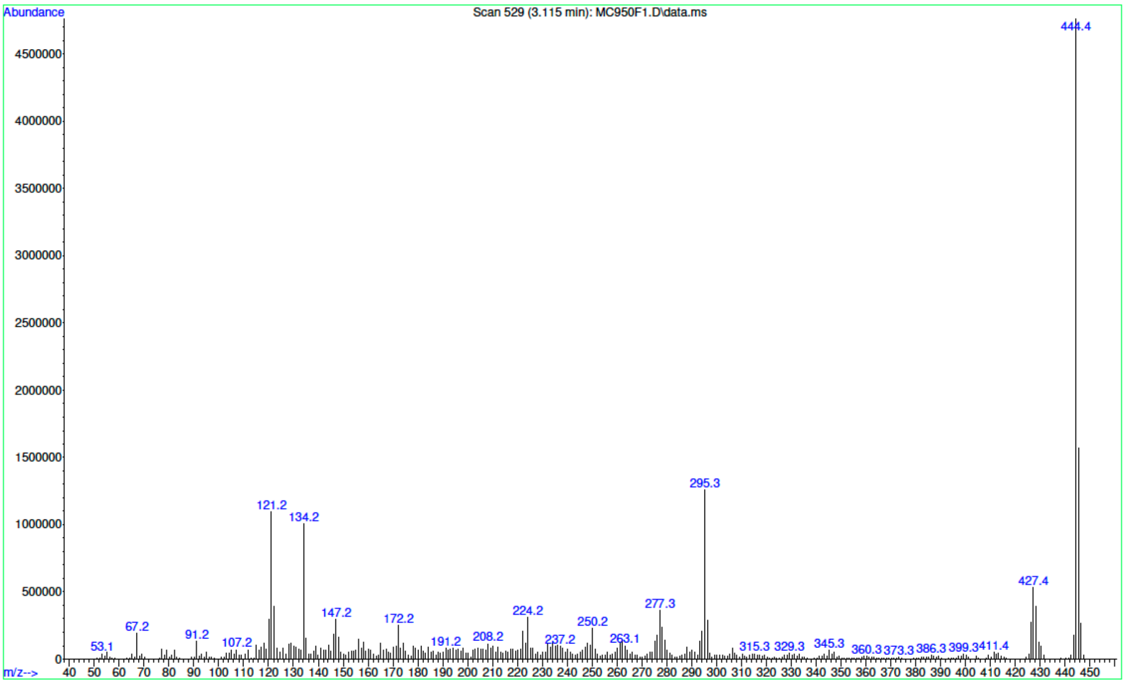
Abundance
Scan 529 (3.115 min): MC950F1.D\data.ms
m/z-->

**Data File**	190_MC950F1_01.d	**Sample Name**	MC950F1
**Sample Type**	Sample	**Position**	Vial 7
**Instrument Name**	Instrument 1	**User Name**	
**Acq Method**	ESI_ACN_75_pos_new.m	**Acquired Time**	2/8/2021 12:57:17 PM (UTC+01:00)
**IRM Calibration Status**	Success	**DA Method**	Defecto_modificado.m
**Comment**			

**Sample Group**		**Info.**	
**User**	MOURAD CHIOUA	**Stream Name**	LC 1
**Acquisition Time (Local)**	2/8/2021 12:57:17 PM (UTC+01:00)	**Acquisition SW Version**	6200 series TOF/6500 series Q-TOF B.08.00 (B8058.3 SP1)
**QTOF Driver Version**	8.00.00	**QTOF Firmware Version**	2.712
**Tune Mass Range Max.**	1700		

Compound Table

Compound Label	RT	Mass	Abund	Formula	Tgt Mass	Diff (ppm)	Hits (DB)
Cpd 1: C29 H36 N2 O2; 1.529	1.529	444.2771	326972	C29 H36 N2 O2	444.2777	-1.41	1

Compound Label	*m/z*	RT	Algorithm	Mass
Cpd 1: C29 H36 N2 O2; 1.529	445.2845	1.529	Find by Formula	444.2771

MS Zoomed Spectrum

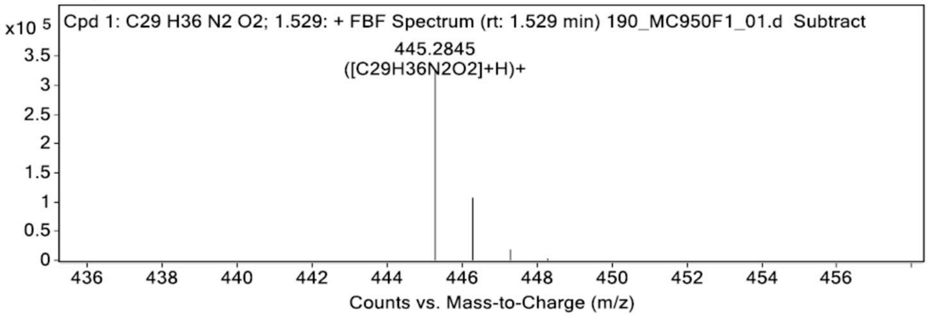

MS Spectrum Peak List

m/z	z	Abund	Formula	Ion
445.2845	1	326971.78	C29H36N2O2	(M+H)+
446.287	1	107501.92	C29H36N2O2	(M+H)+
447.2919	1	18593.35	C29H36N2O2	(M+H)+
448.2907	1	3144.23	C29H36N2O2	(M+H)+

MC5

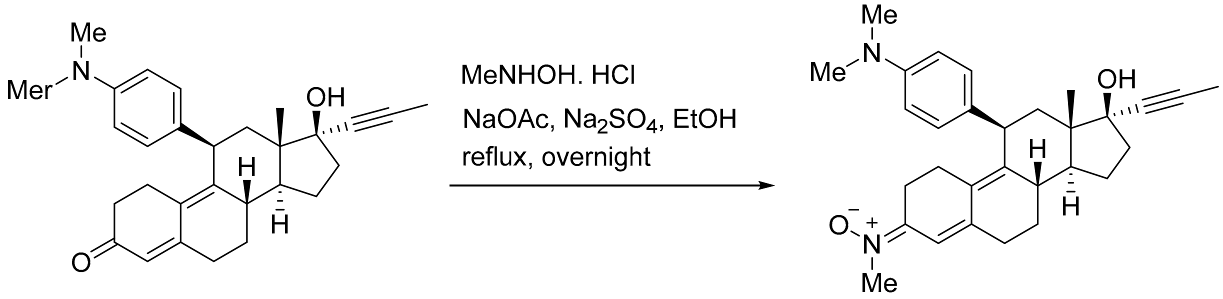

(8*S*,11*R*,13*S*,14*S*,17*S*,*E*)-11-(4-(Dimethylamino)phenyl)-17-hydroxy-*N*,13-dimethyl-17-(prop-1-yn-1-yl)-1,2,6,7,8,11,12,13,14,15,16,17-dodecahydro-3*H*-cyclopenta[α]phenanthren-3-imine oxide (MC5). Following the **General Method for the Synthesis of the Nitrones**, a solution of commercial mifepristone {(8*S*,11*R*,13*S*,14*S*,17*S*)-11-(4-(dimethylamino)phenyl)-17-hydroxy-13-methyl-17-(prop-1-yn-1-yl)-1,2,6,7,8,11,12,13,14,15,16,17-dodecahydro-3*H*-cyclopenta[*a*]phenanthren-3-one} (215 mg, 0.5 mmol), Na_2SO_4 (213 mg, 1.5 mmol, 3 equiv), AcONa (82 mg, 1 mmol, 2 equiv) and *N*-methylhydroxylamine hydrochloride (63 mg, 0.75 mmol, 1.5 equiv) in EtOH (7 mL) was heated at 90 °C for 16 h. Then, the solvent was evaporated and the crude mixture was purified on column chromatography (D/M 0%-50%) to yield compound **MC5** (fraction F2: 9,3 mg, 4%, as a single and pure *E* isomer; fraction F3: 130 mg, 57%, as mixture of *E*/*Z*: 3/2). **(*E*)-MC951**: mp 171-3 ºC; ^{1}H NMR (400 MHz, $CDCl_3$) δ 7.00 (d, J = 8.5 Hz, 2H), 6.68 – 6.59 (m, 2H), 5.99 (s, 1H), 4.28 (d, *J* = 6.7 Hz, 1H), 3.72 (s, 3H), 2.99 (dt, *J* = 17.7, 5.7 Hz, 1H), 2.89 (s, 6H), 2.64 (dt, *J* = 15.1, 5.4 Hz, 1H), 2.52 (q, *J* = 7.8, 4.5 Hz, 3H), 2.42 (s, 1H), 2.35 – 2.14 (m, 3H), 2.11 – 1.89 (m, 2H), 1.88 (s, 3H), 1.76 – 1.59 (m, 2H), 1.47 – 1.25 (m, 1H), 0.51 (s, 3H); ^{13}C NMR (101 MHz, $CDCl_3$) δ 174.0, 148.5, 147.8, 144.1, 143.5, 132.4, 128.9, 127.6, 112.8, 82.5, 82.4, 80.3, 77.4, 77.3, 77.1, 76.8, 50.0, 46.9, 46.0, 40.7, 39.3, 39.2, 38.9, 38.8, 31.8, 28.0, 24.1, 23.7, 23.4, 21.1, 13.8, 3.9. HRMS (ESI_ACN): Calcd. for $[C_{30}H_{38}N_2O_2]^+$: 459,3006. Found 459,3004 $[M + 1]^+$.

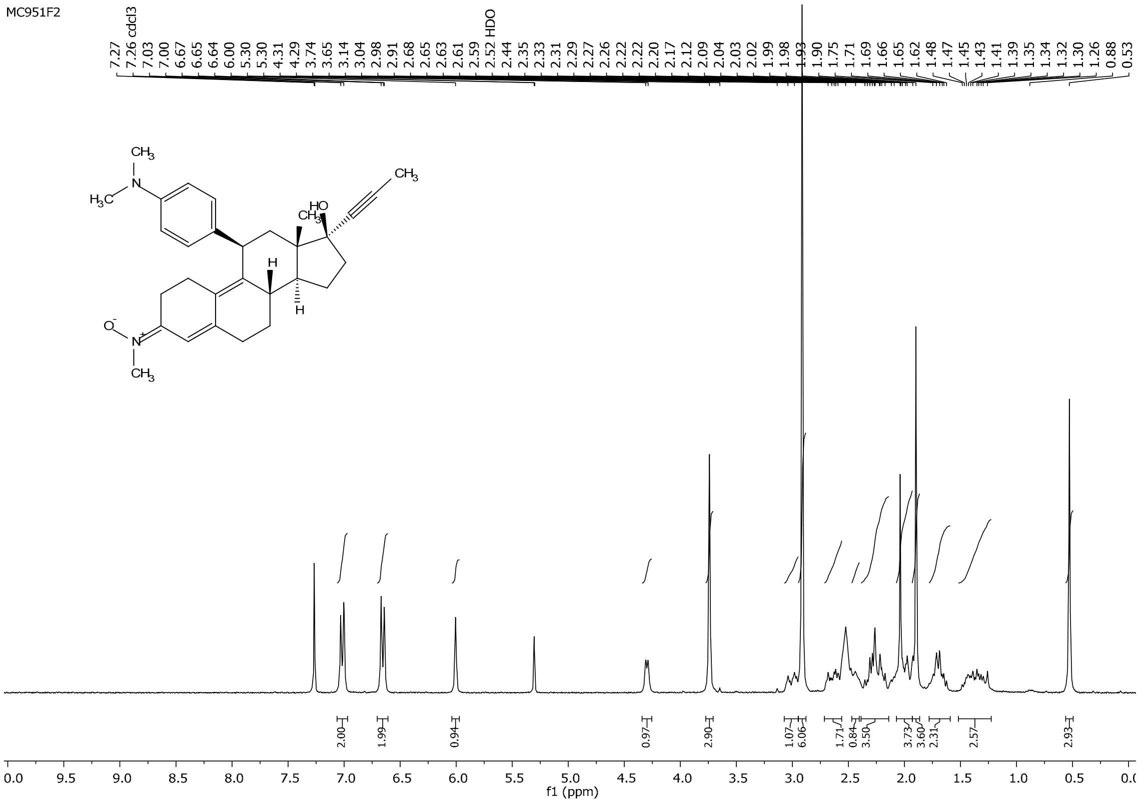
MC951F2
7.27 7.26 cdcl3 7.03 7.00 6.67 6.65 6.64 6.00 5.30 5.30 4.31 4.29 3.74 3.65 3.14 3.04 2.98 2.91 2.68 2.65 2.63 2.61 2.59 2.52 HDO 2.44 2.35 2.33 2.31 2.29 2.27 2.26 2.22 2.22 2.20 2.17 2.12 2.09 2.04 2.03 2.02 1.99 1.98 1.93 1.90 1.75 1.71 1.69 1.66 1.65 1.62 1.48 1.47 1.45 1.43 1.41 1.39 1.35 1.34 1.32 1.30 1.26 0.88 0.53
CH3
N
H3C
HO
CH3
CH3
H
H
O-
N+
CH3
2.00 1.99 0.94 0.97 2.90 1.07 6.06 1.71 0.84 3.50 3.73 3.60 2.31 2.57 2.93
f1 (ppm)

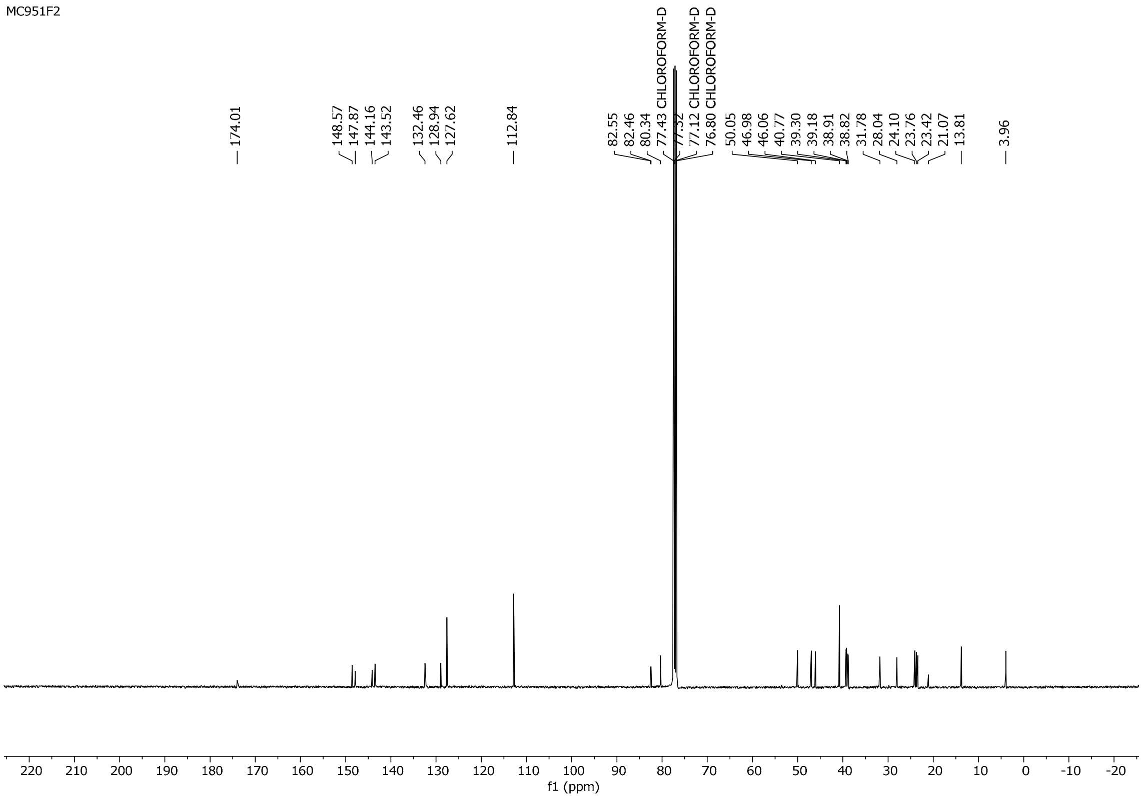
MC951F2
174.01 148.57 147.87 144.16 143.52 132.46 128.94 127.62 112.84 82.55 82.46 80.34 77.43 CHLOROFORM-D 77.32 77.12 CHLOROFORM-D 76.80 CHLOROFORM-D 50.05 46.98 46.06 40.77 39.30 39.18 38.91 38.82 31.78 28.04 24.10 23.76 23.42 21.07 13.81 3.96
f1 (ppm)

**Data File**	193_MC951F2_01.d	**Sample Name**	MC951F2
**Sample Type**	Sample	**Position**	Vial 10
**Instrument Name**	Instrument 1	**User Name**	
**Acq Method**	ESI_ACN_75_pos_new.m	**Acquired Time**	2/8/2021 1:19:14 PM (UTC+01:00)
**IRM Calibration Status**	Success	**DA Method**	Defecto_modificado.m
**Comment**			

**Sample Group**		**Info.**	
**User**	MOURAD CHIOUA	**Stream Name**	LC 1
**Acquisition Time (Local)**	2/8/2021 1:19:14 PM (UTC+01:00)	**Acquisition SW Version**	6200 series TOF/6500 series Q-TOF B.08.00 (B8058.3 SP1)
**QTOF Driver Version**	8.00.00	**QTOF Firmware Version**	2.712
**Tune Mass Range Max.**	1700		

Compound Table

Compound Label	RT	Mass	Abund	Formula	Tgt Mass	Diff (ppm)	Hits (DB)
Cpd 1: C30 H38 N2 O2; 1.361	1.361	458.2929	996004	C30 H38 N2 O2	458.2933	-1.03	1

Compound Label	*m/z*	RT	Algorithm	Mass
Cpd 1: C30 H38 N2 O2; 1.361	459.3004	1.361	Find by Formula	458.2929

MS Zoomed Spectrum

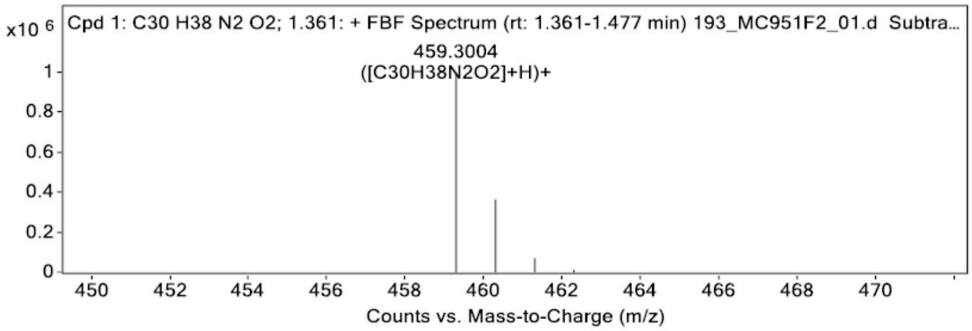

MS Spectrum Peak List

m/z	z	Abund	Formula	Ion
459.3004	1	996004.38	C30H38N2O2	(M+H)+
460.3029	1	362933.97	C30H38N2O2	(M+H)+
461.3057	1	68912.22	C30H38N2O2	(M+H)+
462.3084	1	9300.3	C30H38N2O2	(M+H)+

MC6, MC7

MC6

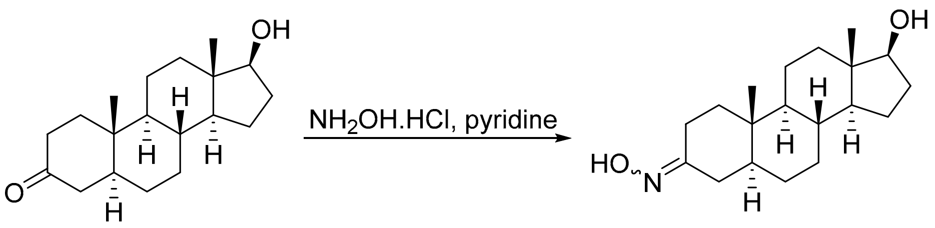

(5*S*,8*R*,9*S*,10*S*,13*S*,14*S*,17*S*)-17-Hydroxy-10,13-dimethylhexadecahydro-3*H*-cyclopenta[*a*]phenanthren-3-one oxime (MC6) [1. Camoutsis, C.; Catsoulacos, P. Formation of bishomoazasteroids by the Beckmann rearrangement. J. *Heterocyclic Chem.* **1988**, *25*, 1617-19; Oka, K.; Hara, S. Nuclear magnetic resonance spectra on syn and anti isomers of steroidal 3-ketoximes. *Chem. Ind.* **1968**, *27*, 911-12] Following the **General Method for the Synthesis of the Oximes**, a solution of commercial stanolone {(5*S*,8*R*,9*S*,10*S*,13*S*,14*S*,17*S*)-17-hydroxy-10,13-dimethylhexadecahydro-3*H*-cyclopenta[*a*]phenanthren-3-one} (290 mg, 1 mmol, 1 equiv), hydroxylamine hydrochloride (104 mg, 1.5 mmol, 1.5 equiv) in pyridine (5 mL) was heated at 90 °C for 2 h. After that time, the solvent was evaporated and the crude mixture was purified on column chromatography (DCM/metahnol 0%-10%) to yield compound **MC6** (white solid, 300 mg, 98%) as an unseparable mixture of isomers in a 1:1 ratio: mp 214-7 ºC; ^{1}H NMR (300 MHz, $CDCl_3$) δ 3.64 [dd, *J*= 9.0, 8.0 Hz, 1H, *H*-C17(OH)], 3.49 (s, 1H, OH), 3.25 (dd, *J*= 18, 6 Hz, H2 , isomer *E*), 3.00 (ddd, *J*= 15, 3.2, 1.4 Hz, H2 , isomer *Z*), 2.33-2.20 (m, 1H), 2.20-1.96 (m, 2H), 1.90-1.79 (m, 3H), 1.75-1.50 (m, 4H), 1.49-1.19 (m, 5H), 1.12-0.75 (m, 5H) , 0.92 (s, 3H), 0.75 (s, 3H), 0.72-0.62 (m, 1H) (the signal for =N-OH was not detected); MS (EI) *m*/*z*: 305 [M, 100]$^{+\cdot}$, 288 [M-OH, 39]$^{+\cdot}$, 272 [M-NOH, 28]$^{+}$. HRMS (ESI+_ACN): Calcd. for $[C_{19}H_{31}NO_2]^+$: 306.2428. Found 306.2413 $[M + 1]^+$.

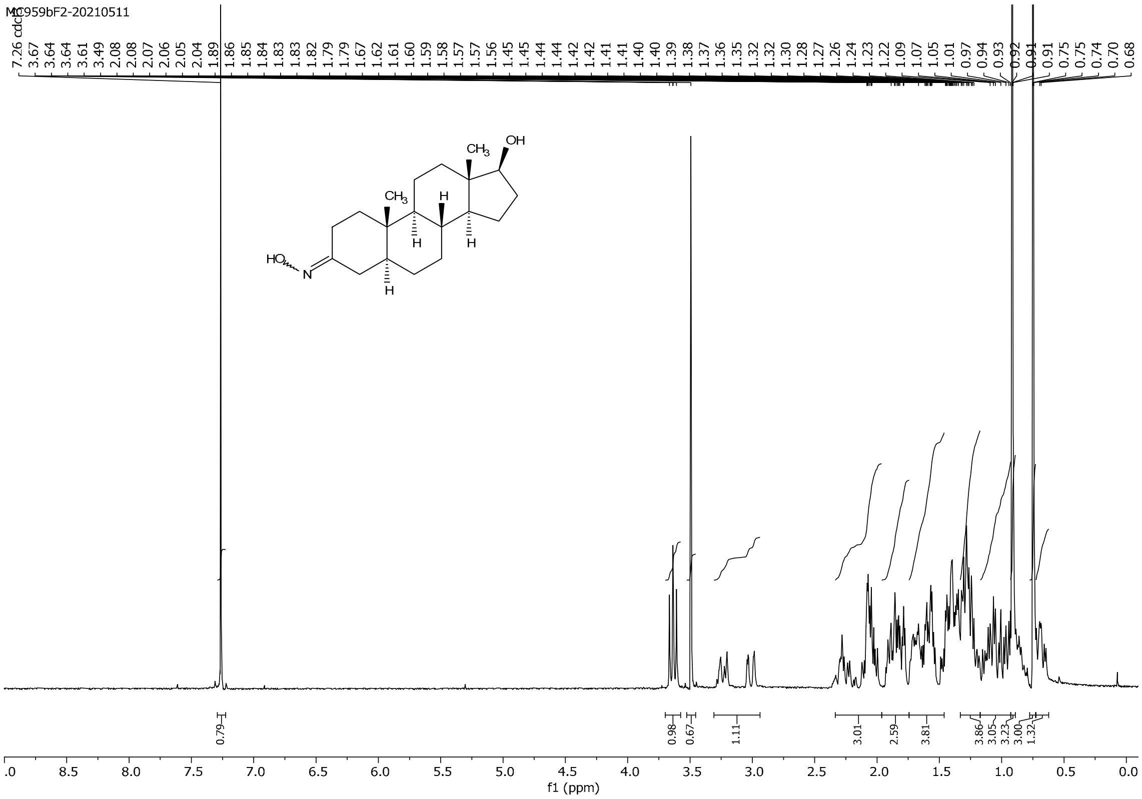

File   :C:\MSDCHEM\1\DATA\2021\MAYO 2021\Snapshot\MC959F2.D
Operator   :
Acquired   : 13 May 2021 10:03   using AcqMethod INYECCION DIRECTA.M
Instrument :   MASAS IMPACTO
Sample Name:
Misc Info : Mourad Chioua (IQOG)
Vial Number: 1

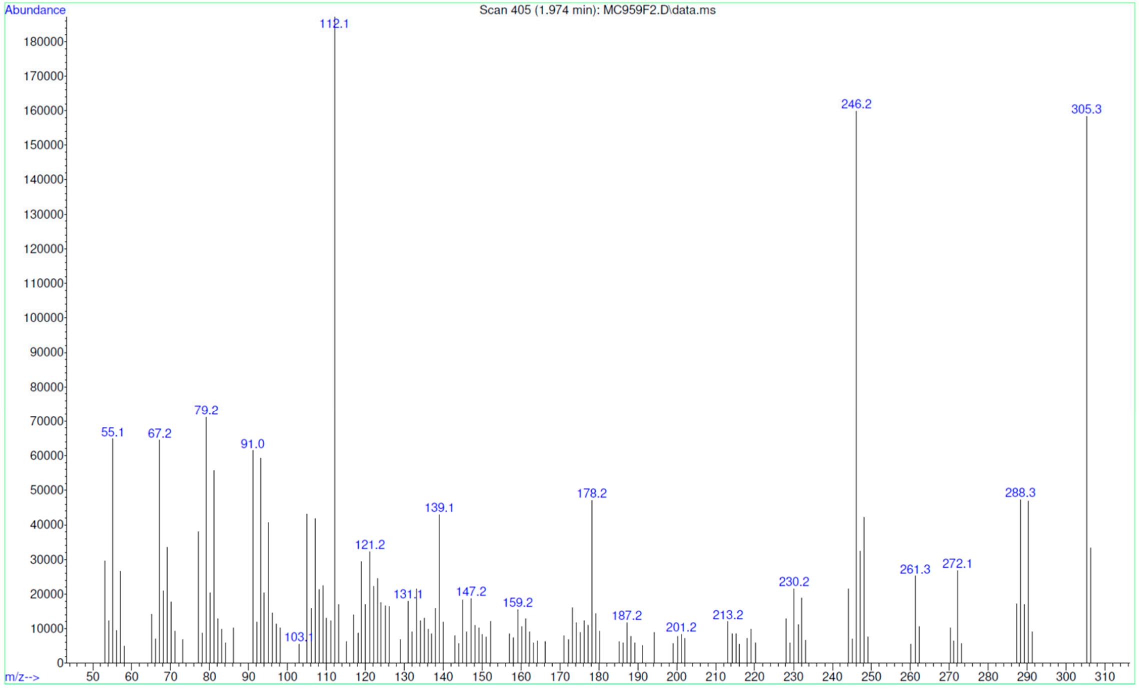

Qualitative Compound Report

**Data File**	478_MC959bF2_01.d	**Sample Name**	MC959bF2
**Sample Type**	Sample	**Position**	Vial 12
**Instrument Name**	Instrument 1	**User Name**	
**Acq Method**	ESI_ACN_75_pos_new.m	**Acquired Time**	5/14/2021 1:38:23 PM (UTC+01:00)
**IRM Calibration Status**	Success	**DA Method**	Defecto_modificado.m
**Comment**			

**Sample Group**		**Info.**	
**User**	MOURAD CHIOUA	**Stream Name**	LC 1
**Acquisition Time (Local)**	5/14/2021 1:38:23 PM (UTC+01:00)	**Acquisition SW Version**	6200 series TOF/6500 series Q-TOF B.08.00 (B8058.3 SP1)
**QTOF Driver Version**	8.00.00	**QTOF Firmware Version**	2.712
**Tune Mass Range Max.**	1700		

Compound Table

Compound Label	RT	Mass	Abund	Formula	Tgt Mass	Diff (ppm)	Hits (DB)
Cpd 1: C19 H31 N O2; 0.269	0.269	305.2341	830015	C19 H31 N O2	305.2355	-4.43	1

Compound Label	*m/z*	RT	Algorithm	Mass
Cpd 1: C19 H31 N O2; 0.269	306.2413	0.269	Find by Formula	305.2341

MS Zoomed Spectrum

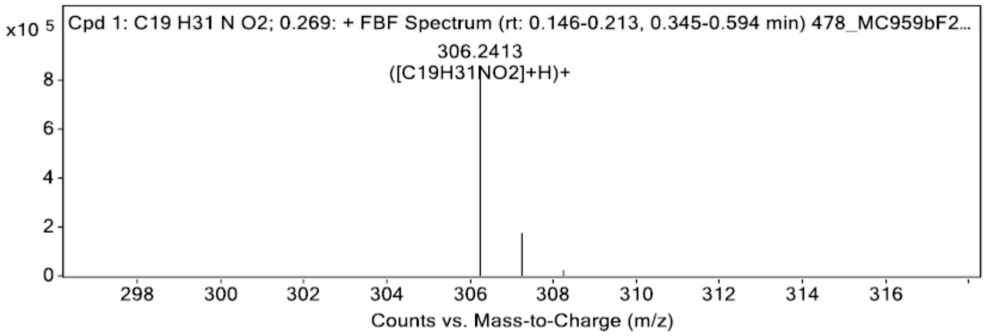

MC7

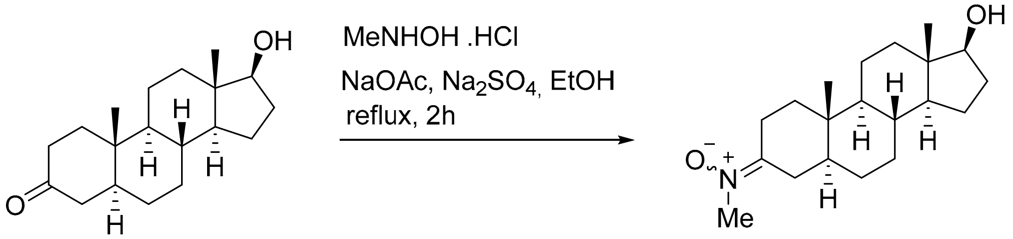

(5*S*,8*R*,9*S*,10*S*,13*S*,14*S*,17*S*)-17-Hydroxy-*N*,10,13-trimethylhexadecahydro-3*H*-cyclopenta[*a*]phenanthren-3-imine oxide (MC7) [Weintraub, P. M.; Tiernan, P. L. Steroidal nitrones. *J. Org. Chem*. **1974**, *39*, 1061-5]. Following the **General Method for the Synthesis of the Nitrones**, a solution of commercial stanolone {(5*S*,8*R*,9*S*,10*S*,13*S*,14*S*,17*S*)-17-hydroxy-10,13-dimethylhexadecahydro-3*H*-cyclopenta[*a*]phenanthren-3-one} (290 mg, 1 mmol), Na_2SO_4 (426 mg, 3 mmol, 3 equiv), AcONa (164 mg, 2 mmol, 2 equiv) and *N*-methylhydroxylamine hydrochloride (84 mg, 1.5 mmol, 1.5 equiv) in EtOH (7 mL) was heated at 90 °C for 16 h. After that time, the solvent was evaporated and the crude mixture was purified on column chromatography (D/M 0%-50%) to yield compound **MC7** (white solid, 212 mg, 65 %) as an unseparable mixture of isomers in a 1/1 ratio: mp 105-7 ºC (lit. [1] 196-8 ºC); ^{1}H NMR (300 MHz, $CDCl_3$) δ 3.67 [s, 3H, =N(O)CH_3], 3.64 (m, 1H, H-C17), 3.31 [dd, *J*= 18.5, 5.3 Hz, 1H, H2 , isomer *E* (*Z*)], 3.22 [m, 1H, H2 , isomer *Z* (*E*)], 2.88 (s, 1H, OH), 2.62-0.65 (m, 21H), 0.75 (s, 3H), 0.69 (s, 3H); MS (EI) *m*/*z*: 319 [M, 100]$^{+\cdot}$, 303 [M-O, 63]$^+$. HRMS (ESI+_ACN): Calcd. for [$C_{20}H_{33}NO_2$]$^+$: 320.2584. Found 320.2582 [M + 1]$^+$.

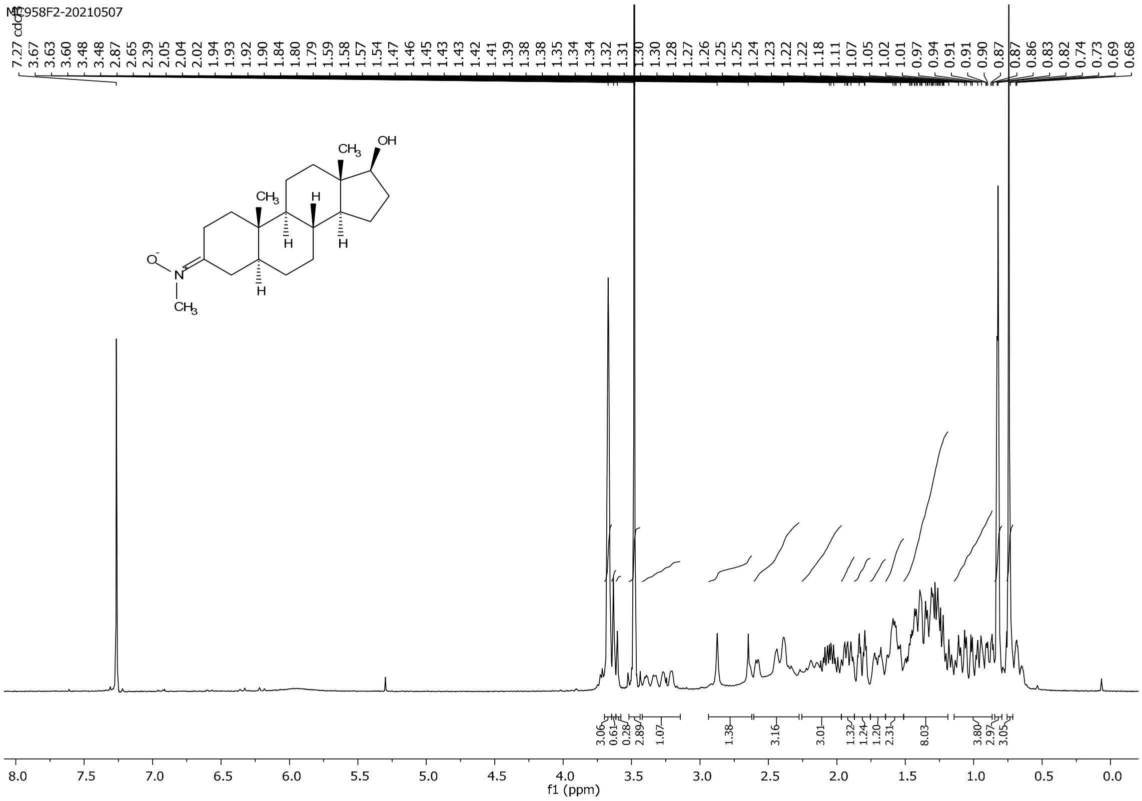

MC958F2-20210507
cdcl3
7.27
3.67
3.63
3.60
3.48
3.48
2.87
2.65
2.39
2.05
2.04
2.02
1.94
1.93
1.92
1.90
1.84
1.80
1.79
1.59
1.58
1.57
1.54
1.47
1.46
1.45
1.43
1.43
1.42
1.41
1.39
1.38
1.38
1.35
1.34
1.34
1.32
1.31
1.30
1.30
1.28
1.27
1.26
1.25
1.25
1.24
1.23
1.22
1.22
1.22
1.18
1.11
1.07
1.05
1.02
1.01
0.97
0.94
0.91
0.91
0.90
0.87
0.86
0.83
0.82
0.74
0.73
0.69
0.68
OH
CH3
CH3
H
H
H
H
O-
N
CH3
3.06
0.61
0.28
2.89
1.07
1.38
3.16
3.01
1.32
1.24
1.20
2.31
8.03
3.80
2.97
3.05
8.0
7.5
7.0
6.5
6.0
5.5
5.0
4.5
4.0
3.5
3.0
2.5
2.0
1.5
1.0
0.5
0.0
f1 (ppm)

```
File        :C:\MSDCHEM\1\DATA\2021\MAYO 2021\Snapshot\MC958F2.D
Operator    :
Acquired    : 13 May 2021   9:42     using AcqMethod INYECCION DIRECTA.M
Instrument :   MASAS IMPACTO
Sample Name:
Misc Info   : Mourad Chioua (IQOG)
Vial Number: 1
```

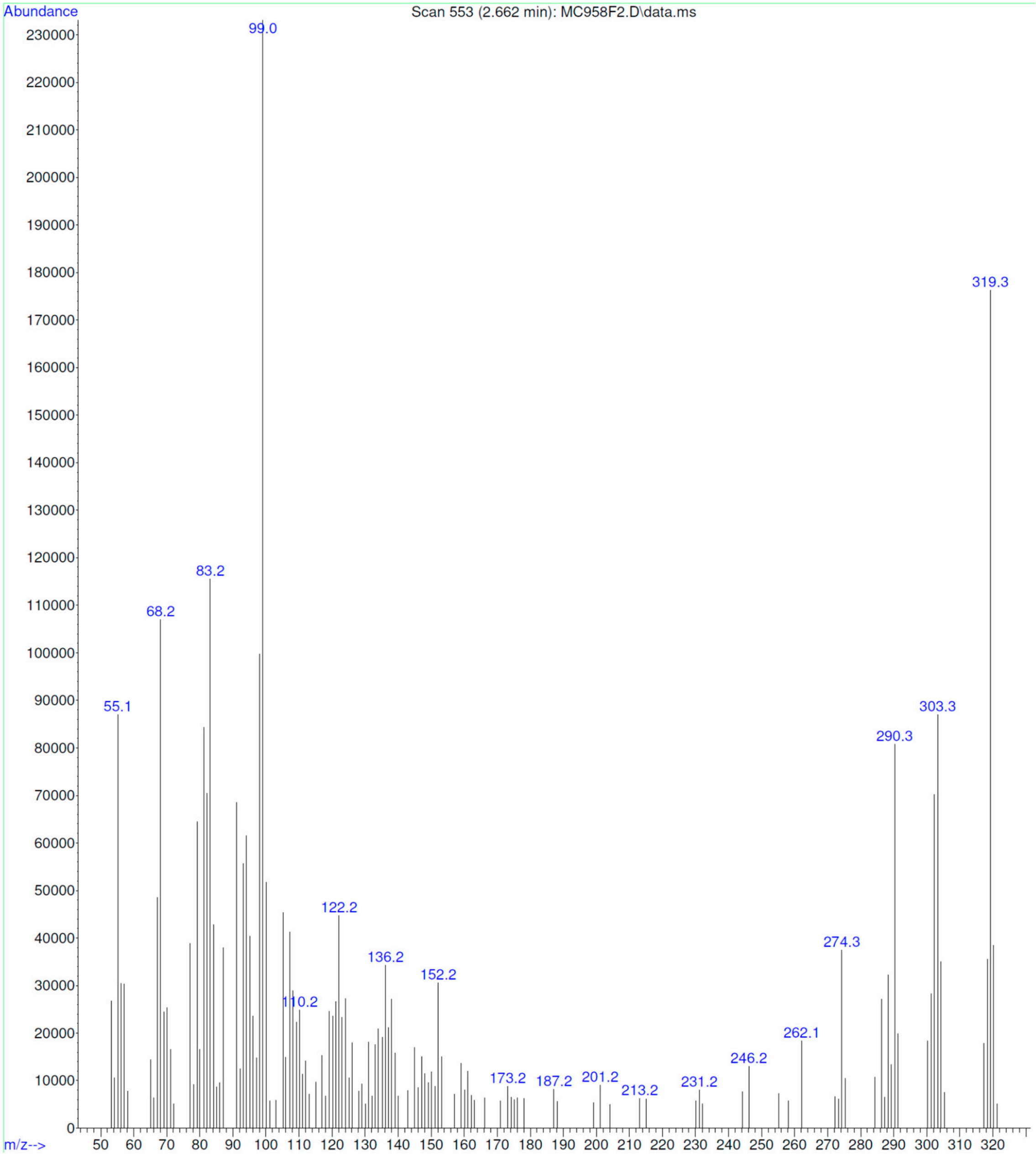

Qualitative Compound Report

**Data File**	477_MC958F2_01.d	**Sample Name**	MC958F2
**Sample Type**	Sample	**Position**	Vial 11
**Instrument Name**	Instrument 1	**User Name**	
**Acq Method**	ESI_ACN_75_pos_new.m	**Acquired Time**	5/14/2021 1:31:04 PM (UTC+01:00)
**IRM Calibration Status**	Success	**DA Method**	Defecto_modificado.m
**Comment**			

**Sample Group**		**Info.**	
**User**	MOURAD CHIOUA	**Stream Name**	LC 1
**Acquisition Time (Local)**	5/14/2021 1:31:04 PM (UTC+01:00)	**Acquisition SW Version**	6200 series TOF/6500 series Q-TOF B.08.00 (B8058.3 SP1)
**QTOF Driver Version**	8.00.00	**QTOF Firmware Version**	2.712
**Tune Mass Range Max.**	1700		

Compound Table

Compound Label	RT	Mass	Abund	Formula	Tgt Mass	Diff (ppm)	Hits (DB)
Cpd 1: C20 H33 N O2; 1.392	1.392	319.2509	467664	C20 H33 N O2	319.2511	-0.71	1

Compound Label	*m/z*	RT	Algorithm	Mass
Cpd 1: C20 H33 N O2; 1.392	320.2582	1.392	Find by Formula	319.2509

MS Zoomed Spectrum

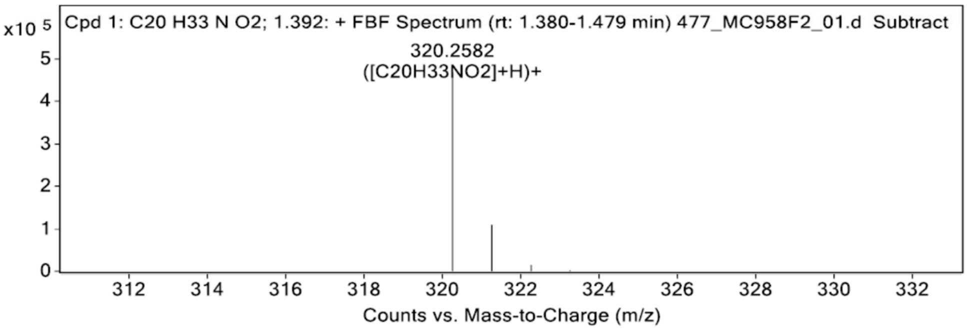

Supplement: Supplementary file 1 [file ijms-26-11506-s001.zip › ijms-3985142-supplementary.pdf]
